# PM6-ML: The Synergy of Semiempirical Quantum Chemistry and Machine Learning Transformed into a Practical Computational Method

**DOI:** 10.1021/acs.jctc.4c01330

**Published:** 2025-01-03

**Authors:** Martin Nováček, Jan Řezáč

**Affiliations:** Institute of Organic Chemistry and Biochemistry, Czech Academy of Sciences, 160 00 Prague, Czech Republic

## Abstract

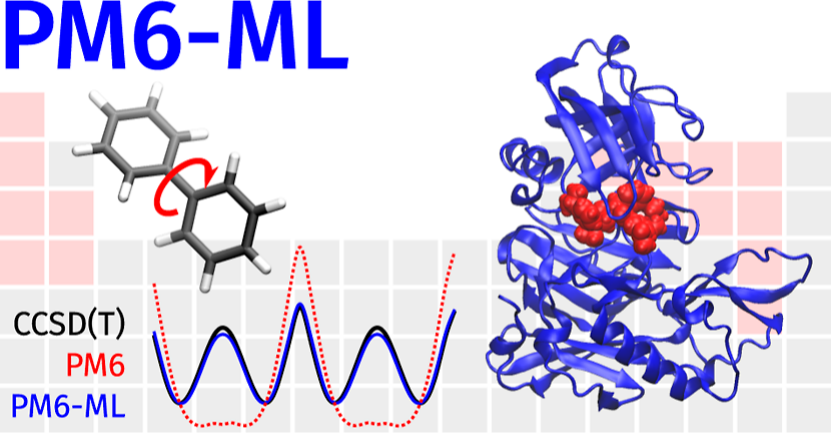

Machine learning (ML) methods offer a promising route to the construction of universal molecular potentials with high accuracy and low computational cost. It is becoming evident that integrating physical principles into these models, or utilizing them in a Δ-ML scheme, significantly enhances their robustness and transferability. This paper introduces PM6-ML, a Δ-ML method that synergizes the semiempirical quantum-mechanical (SQM) method PM6 with a state-of-the-art ML potential applied as a universal correction. The method demonstrates superior performance over standalone SQM and ML approaches and covers a broader chemical space than its predecessors. It is scalable to systems with thousands of atoms, which makes it applicable to large biomolecular systems. Extensive benchmarking confirms PM6-ML’s accuracy and robustness. Its practical application is facilitated by a direct interface to MOPAC. The code and parameters are available at https://github.com/Honza-R/mopac-ml.

## 1 Introduction

Machine learning (ML) methods represent a promising avenue for the construction of universal molecular potentials with high accuracy and favorable computational cost. However, it is becoming increasingly evident that incorporating some physics into the model confers advantages, rendering the model more robust and reducing the amount of data required for training. This approach can be taken to the extreme, whereby machine learning can be applied on top of a complete but approximate computational method and trained to reproduce more accurate calculations. This approach, designated delta-machine learning (Δ-ML), is particularly well-suited for enhancing the precision of computationally inexpensive yet broadly applicable computational chemistry methods.

The same topic can be viewed from an alternative perspective. Semiempirical quantum-mechanical (SQM) methods^1,2^ offer a distinctive combination of universal applicability, rooted in a solid physical background, and of favorable computational efficiency. However, this efficiency is achieved by introducing approximations that limit the accuracy of the method. We, along with other researchers, have been engaged in efforts to address some of these limitations, particularly those related to the description of noncovalent interactions (NCIs),^3−8^ with the goal of developing methodology applicable to large systems such as biomolecules.^9−11^ These corrections, along with novel methods incorporating them, have led to a notable advancement over the previous state of the art. However, there remain challenges that cannot be resolved through this approach.

This work builds on our experience with the development of corrections for SQM methods. Instead of using additional corrections addressing specific phenomena, we have constructed a Δ-ML model combining PM6,^12^ an universal SQM method, with state-of-the-art ML potential applied as correction and trained to reproduce high-quality DFT calculations. The resulting method, named PM6-ML, is shown to outperform both standalone SQM and ML methods. In comparison to earlier SQM-based Δ-ML methods, this new approach offers greater accuracy and, for the first time, covers a wider chemical space, what renders it applicable to real-world chemical problems.

The selection of PM6 as the baseline method was driven by its inherent properties and the availability of a linear-scaling implementation,^13^ which allows it to be applied to very large molecular systems. PM6 is a classical SQM method based on the NDDO (neglect of diatomic differential overlap) approximation,^14^ which is universally applicable to a broad chemical space.^12^ It served as the main platform for the development of our earlier corrections for noncovalent interactions, and with these, as PM6-D3H4X,^4,5^ it is one of the most accurate SQM methods in our primary area of applications, namely biomolecules.^9,10^ These findings were validated by extensive benchmarking, which simultaneously revealed the method’s most significant deficiencies. In particular, the PM6-D3H4X SQM method exhibited poor description of noncovalent interactions at very short distances^15,16^ and significant errors in relative energies of conformers,^17^ which are limitations shared by other SQM methods. Despite our best efforts, we were unable to identify a satisfactory solution to these issues through additional corrections or reparametrization of PM6 itself. This ultimately led us to pursue the ML correction as a potential solution.

Since our goal is to reproduce high quality reference data, the ML potential used as a correction must be able to achieve high accuracy, but it must also be data efficient, since the reference computations themselves are quite demanding. Both requirements are met by the equivariant transformer (ET) models, which represent the current state of the art in ML potentials. Among the few implementations of ETs available at the time we started this project, we had chosen the TorchMD-NET/ET potential^18^ because it had already demonstrated its applicability as a stand-alone ML potential covering the same chemical space we were targeting (note that the potential is labeled “TorchMD-NET/ET” for the purpose of distinguishing it from the TorchMD-NET framework, which also implements other models).

The primary data utilized for the training of the correction is the SPICE database,^19^ which offers comprehensive coverage of biomolecules, organic compounds, and ions comprising 15 elements (H, C, N, O, P, S, F–I, Li–K, Mg, Ca). This represents a significant advantage over the preceding SQM-based Δ-ML approaches,^20,21^ which are applicable to only four elements (H, C, N, O). Another advantage of the SPICE database is that it has been computed at a very high level using one of the top-performing DFT functionals, ωB97M-D3BJ,^22^ in a large def2-TZVPPD basis set.^23^ We complemented the SPICE data set by additional systems covering noncovalent interactions taken from the NCIAtlas database.^16,24−27^

The development of the method is supported by extensive benchmarking, including comparisons with previous Δ-ML approaches, a range of SQM methods, and multiple standalone ML potentials. The methods were evaluated using a diverse collection of data sets that encompassed the intended applications to organic and biomolecular systems. Furthermore, selected methods were evaluated in a realistic scenario derived from previous research on protein–ligand interactions.

The results presented here demonstrate that the PM6-ML method outperforms both the components it is constructed from—namely, SQM calculations or machine learning alone. The machine learning is able to correct errors in the SQM calculations with unprecedented accuracy. Conversely, the solid physical basis lends the resulting method robustness, which is difficult to achieve with machine learning only. This is demonstrated mainly by the excellent transferability from the small systems used in the training to much larger ones that are important for practical applications.

In order to facilitate the use of PM6-ML, we have implemented a direct interface between the ML correction and MOPAC, which is the leading software in the field of SQM calculations. The code, as well as the parameters for the ML model, are available at https://github.com/Honza-R/mopac-ml.

## 2 Methods

### 2.1 PM6 and Corrections for Noncovalent Interactions

The foundation of PM6-ML is the classical semiempirical method PM6, which is based on the NDDO approximation. There were two primary reasons for utilizing a method from this class: first, these methods are highly robust, exhibiting no convergence issues in large systems, which is a common occurrence in density functional tight binding methods. Second, when computed in the MOPAC software, they can be coupled with the MOZYME linear scaling algorithm,^13^ which makes them faster than any competing method in very large systems (thousands of atoms). Moreover, we have extensive experience with PM6 from our previous work.

PM6-ML is intended as a replacement for the empirical corrections for SQM methods that we developed previously. It will therefore be compared to the most recent version of PM6 with corrections for London dispersion, hydrogen, and halogen bonds, PM6-D3H4X.^4,5^ Specifically, the H4 and X corrections, with parameters updated in refs (24 and 26), and the additional repulsive correction introduced in ref (16), are employed throughout the paper. This method is designated PM6-D3H4X′ to differentiate it from the default version.

All the PM6 calculations presented in this work were conducted using MOPAC.^28^

### 2.2 PM6-ML Correction

The PM6-ML method combines unmodified PM6, the D3 dispersion correction, and the machine learning potential, repurposed to serve as a short-ranged correction for the remaining errors. The ML correction (Δ*E*_ML_) is trained to reproduce the difference between the DFT reference and PM6, both with the same D3 dispersion correction (as parametrized for the DFT functional used) which therefore cancels out and does not enter the Δ*E*_ML_ term. The dispersion correction Δ*E*_D3_ is then added back, so that the final PM6-ML energy is assembled as
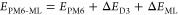
1

The Δ*E*_ML_ correction is based on the TorchMD-NET/ET ML potential, as detailed in ref (18). This potential employs the equivariant transformer architecture,^29,30^ affording it significant advantages. The appropriate handling of spatial symmetries enables the training of more robust potentials with only moderate requirements on the size of the training data set. In contrast to the original setup, which was intended as a standalone potential, we reduce the radial cutoff defining the environment of each atom to 5 Å and use only a single atom type for each element, regardless of the charge of the atom. This is due to the fact that the electrostatics is handled by the underlying SQM calculation. Also, in cases where a correction is required at the short-range, the charge information can be inferred from the environment of the atom. Finally, the original TorchMD-NET/ET model exhibited a discontinuity in the potential at the cutoff distance. This was fixed in collaboration with the authors of the TorchMD-NET software (see the Acknowledgments) and all the models presented here are free of this error.

The D3 dispersion correction in the ωB97M-D3BJ functional^22^ employs the Becke–Johnson damping with parameters *s*_8_ = 0.3908, *a*_1_ = 0.566, and *a*_2_ = 3.128, with these values being utilized here without modification. In addition, the three-body dispersion term is included in the calculation of Δ*E*_D3_, which is not used in the ωB97M-D3BJ functional. This term is negligible in the small molecules used for training, but it should improve the description of larger systems where PM6-ML will likely be used.

### 2.3 Training Data

The training data were obtained from two sources—the SPICE^19^ and the NonCovalent Interaction Atlas^16,24−27^ (NCIAtlas) databases. The SPICE database (version 1.1.2), comprising approximately 1.1 million conformations of small molecules (including peptides, amino acids, drug-like molecules, and ions) and their noncovalent complexes, serves as the backbone of the training data. To enhance the representation of a broader range of noncovalent interactions, the NCIAtlas data sets were further added to the training data. The NCIAtlas is comprised of seven data sets that map disparate classes of noncovalent interactions within an expanded chemical space, and it provides multiple points along the dissociation curve of each complex. From each of these data sets, 50 systems were removed for subsequent use in the validation data set (see Section 2.5 for details). Additionally, systems that fell outside the PM6-ML chemical space were excluded. The final composition of the training data is presented in Table 1.

**Table 1:** Training Data Employed in the Development of PM6-ML Comprises a Combination of the SPICE and NCIAtlas Databases

database	data set	molecules	conformations
SPICE	dipeptides	677	33,850
	solvated amino acids	26	1300
	DES370K	3864	364,376
	PubChem	14,643	731,856
	ion pairs	28	1426
NCIAtlas	D1200	752	752
	D442 × 10	230	2300
	HB300SPX × 10	250	2500
	HB375 × 10	325	3250
	IHB100 × 10	50	500
	Rep739 × 5	504	2520
	SH250 × 10	128	1280
total		21,477	1,145,910

The methods presented here are trained to reproduce the reference DFT atomization energies, Δ_at_*E*_DFT-D3_. Furthermore, the associated DFT gradients are also used in the training, providing a substantial additional data that characterize the potential energy surface of the molecules. The SPICE database^19^ provides both the energies and gradients computed at the ωB97M-D3BJ/def2-TZVPPD level.^22^ The NCIAtlas data sets were calculated at the same level in Orca, version 5.0.3.^31,32^ It should be noted that the ωB97M DFT functional exists in several variants without and with different dispersion corrections, among which the ωB97M-D3BJ version^22^ provides an excellent description of noncovalent interactions^22,27^ while using a dispersion correction that can be easily included in the PM6-ML model.

The PM6-ML correction is trained to reproduce the difference between the DFT and PM6 atomization energies and gradients. For this purpose, the whole training set was recalculated with PM6 in MOPAC. The training data are then constructed as a difference between the DFT and PM6 atomization energies. Since the D3 dispersion correction is already included in the DFT results, the same D3 correction is added to the PM6 side (see the previous section, denoted as PM6-D3) prior this subtraction. The quantity used for the training of the PM6-ML correction, denoted as Δ*E*_train_, is thus defined as
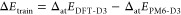
2and its gradient is constructed in the same way.

ML potentials that use molecular geometry as their only input have difficulty handling charged molecules. This is not an issue in the case of PM6-ML, where charge information is included in both the reference DFT and PM6 calculations. Here we evaluate the atomization energies with respect to neutral atoms, so the ionization energy or electron affinity is included in both Δ_at_*E*_DFT-D3_ and Δ_at_*E*_PM6-D3_. Most of it is then subtracted when Δ*E*_train_ is formed (eq 2), and only the difference enters the training data as it is supposed to. In the standalone TorchMDNet/ET potential, we use the approach proposed by the authors of the method. There, the atomization energy is calculated with respect to atoms that retain the formal charge they had in the molecule, which ensures the same number of electrons on both sides of the atomization reaction. In contrast to the original model, we do not employ separate atom types for charged atoms because we wanted to construct only a proof-of-concept model with the same set of parameters as PM6-ML, which is not intended for practical applications. This choice affects the ability of the model to accurately predict absolute atomic energies, but does not affect relative quantities such as interaction or conformational energies discussed in this paper.

### 2.4 Training Procedure

The PM6-ML correction, as well as a reparametrization of the standalone TorchMD-NET/ET potential (see Section 2.7 below), had been trained using the “torchmd-train”’ tool from the TorchMD-NET project.^33^ This tool employs the “Trainer” module of PyTorch^34^ to perform the optimization of the model. The TorchMD-NET/ET architecture consists of three parts: the embedding layer, a series of update layers, and an output network. First, the embedding layer encodes the neighborhood of each atom into a feature vector. Then, the update layers combine the features of the interacting atoms and update this representation. Based on the final feature vector, the output network then predicts the atomic contribution to the energy. The models in this work use the embedding size of 128, a total of 6 update layers with 8 attention heads and a set of 64 trainable radial basis functions each, and an output network consisting of two gated equivariant blocks.

The hyperparameters utilized for training are derived from those used to train the original TorchMD-NET/ET model on the SPICE data set, as provided in the TorchMD-NET GitHub repository.^33^ However, a few modifications have been made: The majority of the training was performed on RTX 3090 graphics cards, which limited the batch size to 62. The upper cutoff was reduced to 5 Å, as the model is mainly meant to correct shorter-range errors. For both the PM6-ML correction and the standalone ML potential, 40 models were trained starting from different randomized initial parameters (keeping all the hyperparameters but the random seed the same).

All models were trained until convergence, as defined by the hyperparameters. Furthermore, we verified that continuing the training beyond this point did not result in any noticeable improvements. It is important to note, however, that the quality of the trained models exhibited significant variation depending on the initial conditions (defined by the random seed used to generate the starting point). Consequently, the performance of all resulting models was subsequently analyzed in more detail, and the best candidates were selected as described in the Results and Discussion, Section 3.1.

### 2.5 Validation Data Sets

The PM6-ML method is benchmarked and compared to other Δ-ML, SQM, and ML approaches using multiple validation data sets covering its primary area of applications. These include noncovalent interactions and conformation energies of organic compounds and biomolecules.

#### 2.5.1 Noncovalent Interactions

The first part of the validation set comprises subsets of all the NCIAtlas data sets, specifically D442, HB375, HB300SPX, R739, SH250, and IHB100, which have been excluded from the training set. It should be noted that, in contrast to the training phase, in some steps of the validation only the equilibrium geometries are employed. This is indicated by dropping the suffix “×···” from the names of the data sets. For each of the aforementioned data sets, the validation subset is constructed by taking the predefined subset of the 50 most diverse systems (obtained by a clustering analysis, as reported in the original publications) and removing systems containing elements outside the PM6-ML chemical space. This results in a reduction of the number of systems to 26 in D442, 22 in R739, and 43 in SH250. These data sets facilitate the interpretation of the results in terms of different classes of noncovalent interactions and the chemical composition of the systems. In some tests, these data sets had been complemented by the widely used, but less diverse S66 set. In all these data sets, the benchmark interaction energies were computed at the coupled cluster with single, double, and perturbative triple excitations [CCSD(T)] level and extrapolated to the complete basis set (CBS) limit. The interaction energies are computed on the fixed structure of the complex, and thus do not include the deformation energy.

#### 2.5.2 Noncovalent Interactions in Large Systems

As the developed method is intended for applications to large molecular systems, it is essential to validate its accuracy in such context. To this end, we employ a series of data sets of increasing size. First, for the widely used L7 and S12L data sets,^35,36^ we utilize the highest-quality benchmark interaction energies, computed using domain-based local pair natural orbital coupled clusters [DLPNO-CCSD(T)], extrapolated to the complete basis set (CBS) limit.^37^ This benchmark is only available for six systems from the S12L set (2a, 2b, 4a, 5a, 6a, and 7b), and these are the only ones used. The systems in question range in size from 72 to 177 atoms. Second, the PLA15 set, as outlined in ref (9), is employed, comprising 15 protein–ligand complex models. There, the DLPNO-CSCD(T) benchmark is constructed from fragment-based calculations, and the systems comprise 283 to 584 atoms. In certain instances, we also examine these fragments, designated as PLF547 data setset, independently. Finally, we assess the performance of PM6-ML in larger protein–ligand complex models (up to 1000 atoms) from the PL-REX data set,^11^ for which DFT calculations are available (136 systems, after zinc-containing complexes had been excluded). These employ a DFT functional similar to the one utilized for PM6-ML training, namely ωB97X-D3BJ, but with a more compact DZVP-DFT basis set^38^ (which had been validated to perform exceptionally well for noncovalent interactions^39^).

#### 2.5.3 Conformation Energies and Torsional Profiles

Another important domain in which the Δ-ML approach can offer substantial enhancements to SQM methods is in the calculation of relative energies of conformers. This is validated in multiple established benchmark data sets. In this context, the relative energies are evaluated with respect to the lowest-energy minimum of each molecule. The MPCONF196 set comprises small peptides and peptidic macrocycles, with conformation energies computed at the DLPNO-CCSD(T) level.^17^ The Amino20 × 4 set, obtained from the GMTKN55 database,^40^ comprises selected conformers of biogenic amino acids computed at the CCSD(T)-F12/CBS level. The SCONF data set, drawn from the same database,^40^ features conformers of sugars, a particularly challenging problem for SQM methods. These data sets of optimized geometries of low-energy conformers are complemented by a sampling of the conformations of a complex drug-like molecule from a room-temperature molecular dynamics simulation from which the snapshots were not further optimized. Specifically, we use the macrocyclic inhibitor of the BACE1 protease from the PL-REX data set, PDB code 5QD5. The structures were obtained from 100 independent short MD simulations at 300 K starting from randomized initial conformations. The simulations used the Amber GAFF2 force field^41^ and the IGB7 solvent model.^42^

Finally, we examine the source of the errors in the conformation energies by analyzing CCSD(T)/CBS torsional profiles of diverse drug-like molecules from the data set of Sellers^43^ (denoted “Torsions” in the remainder of the text).

#### 2.5.4 Evaluation of the Results

The primary measure of the performance of the studied methods in the validation data sets is the root-mean-square error (RMSE). When assessing multiple data sets simultaneously, the RMSE is evaluated in each of them and averaged. In some cases, it is more useful to discuss relative error, which we define as the RMSE divided by the average magnitude of the studied quantity at the benchmark level and express in percent. Additional statistical measures are available in the outputs provided in the Supporting Information.

### 2.6 SQM and DFT Methods Included in the Benchmarking

For the purpose of comparison with earlier SQM approaches, a selection of these was incorporated into the benchmarking process. First, we utilize PM6 without the additional corrections.^12^ Second, PM6 is employed with the most recent corrections for noncovalent interactions, described above and denoted PM6-D3H4X′. Third, PM7 is included, which incorporates analogous corrections for dispersion and hydrogen bonding.^7^ However, it has already been demonstrated to be unsuitable for the application to larger systems.^44^ The calculations were performed using MOPAC^28^ with the D3H4X′ corrections added using the Cuby framework.^45^ Finally, the extended tight binding method GFN2-xTB was also tested, as this represents a different family of SQM methods and is known to perform well in a wide range of benchmarks.^8^

The PM6-ML method had been trained on DFT-D3 reference data; however, the majority of the validation data sets had been computed at a higher level. It is thus necessary to benchmark also the DFT method used to generate the training set with respect to the high-accuracy QM reference. The validation sets were thus recalculated using the same DFT setup, employing the ωB97M-D3BJ functional^22^ and the def2-TZVPPD basis set.^23^ The aforementioned calculations were performed using Orca, version 5.0.3.^31,32^

### 2.7 Δ-ML and ML Methods Included in the Benchmarking

#### 2.7.1 TorchMD-NET/ET

First, in order to enable a comparison between our Δ-ML approach and a pure ML potential with the same setup, the TorchMD-NET/ET model was reparametrized with the same hyperparameters that were used to develop PM6-ML on the same training set. Analogously, the most accurate yet well-balanced model was selected from among the 40 candidates. The resulting model demonstrated superior performance compared to the published models trained on the SPICE data set.^46^ Additionally, the original model fails in large systems because of internal limits of the number of atoms defining the atomic environment. Consequently, we have chosen to utilize only our reparametrization in the benchmarking.

#### 2.7.2 AIMNet2^47^

AIMNet2 is a machine learning potential that incorporates physics-based components, specifically separate terms for electrostatics and London dispersion. It encompasses a relatively broad chemical space. These properties render it a viable candidate for applications to large biomolecular systems. A variety of parameter sets are available for use. In this study, we employ the variant that was trained on the higher-level reference data, ωB97M-D3 DFT calculations, in the ensemble version (averaging over the ensemble of models) which is supposedly the best parameter set available. The code and parameters were downloaded from the AIMNet2 GitHub repository.^48^

#### 2.7.3 MACE-OFF23^49^

MACE-OFF23 is a very recent equivariant message-passing ML potential covering sufficiently large chemical space. However, it is constrained to neutral molecules, which renders it applicable only to a subset of the benchmarks presented in this paper. MACE-OFF23 had been trained on a data set that was primarily derived from the SPICE database, which has also been utilized in this work. The calculations were performed using software^50,51^ and model obtained from the respective GitHub repositories.^52,53^ In this paper, we consistently use the most accurate MACE-OFF23 model labeled “large”.

Other ML methods either do not provide a general model that can be readily used, do not cover the chemical space of our benchmarks, or are not freely available. The latter applies, for example, to the ML and Δ-ML methods described in ref (54), which would be interesting to include in our comparison, but access to them is limited.

### 2.8 Software Implementation

For the development and testing of PM6-ML, we interfaced the TorchMD-NET model to the Cuby framework,^45,56^ which already provides an interface to PM6 calculations in MOPAC and can combine arbitrary methods. The D3 dispersion correction is also provided by Cuby. This implementation is also useful for benchmarking, as it provides access to a wide range of predefined data sets and automates the calculations on them.^57^ It is therefore the reference implementation used in the development of the method and all the results presented here had been computed using this code. The latest version of Cuby includes this interface, and an example input for performing PM6-ML calculations is provided in the documentation at http://cuby4.molecular.cz/interface_torchmdnet.html.

To simplify the use of PM6-ML for existing MOPAC users and to provide access to all the functionality of MOPAC, we also developed a direct interface between MOPAC (written in Fortran) and the code implementing the ML correction (written in Python and using PyTorch as the ML backend) with D3 dispersion provided by simple-dftd3 library.^58^ It consists of a wrapper layer that initializes the ML model and passes control to a modified version of MOPAC, which can then request the computation of the ML correction whenever the SQM energy or gradient is evaluated. This code is available in a GitHub repository https://github.com/Honza-R/mopac-ml. It was tested to closely reproduce the results in the validation data sets introduced above (Section 2.5).

## 3 Results and Discussion

### 3.1 PM6-ML Training and Model Selection

A total of 40 models were trained to convergence for both the TorchMD-NET/ET and PM6-ML methods, initiating the training process from different, randomly generated initial conditions. The considerable variability in the outcomes necessitated a more comprehensive examination.

This variance is evident in the final value of the loss function (the error measure minimized in the training), with some models exhibiting significantly superior performance compared to others. It became evident that only a select few of the 40 models should be chosen for the final use. However, employing training loss as the sole criterion for model selection would not yield the optimal method for practical applications. The composition of the training set is significantly biased (over 60% of the data comprises drug-like molecules sourced from PubChem), and the overall error does not guarantee that the method will perform equally well in cases with lower representation in the training set.

Therefore, an additional metric was evaluated, reflecting both the accuracy of the model and the balance of the description of the different subclasses of the training set. A root-mean-square error (RMSE) was calculated for each subset of the training data, as detailed in Table 1. Furthermore, to enhance the model’s generalizability to larger systems beyond those included in the training set, we assessed also the RMSE for the interaction energies in the L7 and S12L data sets from the validation set. The final error measure used for the selection of the optimal models is a product of the aforementioned RMSEs. In general, this RMSE product correlates well with the overall loss function; however, it alters the ordering of the top-performing models, favoring those with greater balance.

A concise statistical comparison between the best models and the median is presented in Table 3, which also illustrates the superiority of the Δ-ML approach, PM6-ML, over the pure ML model. The individual results for all models are provided in the Supporting Information, Tables S5–S10. All the results presented in the remaining part of the paper were computed with the best model selected using the aforementioned procedure. For PM6-ML, it is the model labeled “seed8” in the tables in the Supporting Information. This model will also be released along with the code implementing the PM6-ML method. For TorchMD-NET/ET, it is the model denoted “seed25”.

**Table 2:** Δ-ML and ML Methods Used or Discussed in the Paper

Δ-ML	ref	elements	notes
PM6-ML	this work	15: H, C, N, O, P, S, F–I, Li–K, Mg, Ca	SQM + equivariant transformer
AIQM1	(20)	4: H, C, N, O	SQM + ANI NN
QD-π	(21)	4: H, C, N, O	DFTB3 + ML
QDπ-2	(55)	12: H, C, N, O, P, S, F–I, Na, K	GFN2-xTB + ML

ML	ref	elements	notes
TorchMD-NET/ET	(18), this work	15: H, C, N, O, P, S, F–I, Li–K, Mg, Ca	equivariant transformer
AIMNet2	(47)	14: H, B, C, N, O, F–I, P, S, Si, As, Se	ML + Coulomb + D3
MACE-OFF23	(49)	10: H, C, N, O, F, P, S, Cl, Br, I	ML, neutral molecules only

**Table 3:** Metric Used to Select the Final Model, a Product of Root Mean Square Errors Computed in the Individual Subsets of the Training Set, and the Final Value of the Loss Function Used in the Training, for Five Best Models and the Median of All the 40 Models Trained

method	model	RMSE product	training loss
PM6-ML	1 (seed8)	0.09	108.93
	2 (seed15)	0.27	110.71
	3 (seed21)	0.28	113.92
	4 (seed17)	0.31	117.30
	5 (seed3)	0.39	114.18
	median	1.09	116.16
TorchMD-NET/ET	1 (seed25)	840	184.52
	2 (seed2)	879	171.22
	3 (seed19)	1126	163.28
	4 (seed31)	1361	178.67
	5 (seed30)	1449	174.02
	median	20,788	185.81

### 3.2 Comparison to Earlier SQM/Δ-ML Approaches

**Figure 1:**
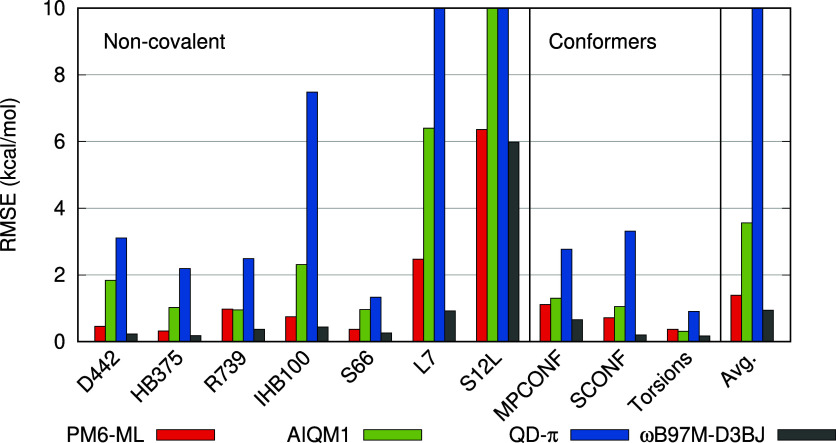
Comparison of PM6-ML to the earlier SQM Δ-ML methods, AIQM1 and QD-π, and to the ωB97M-D3BJ DFT calculations used for training PM6-ML. Subsets of the validation data sets containing only H, C, N and O elements were used. RMSE in kcal/mol.

First, we compare PM6-ML with the two other available Δ-ML methods based on SQM calculations, AIQM1^20^ and QD-π.^21^ They are both applicable to molecules with only four elements (H, C, N and O), which limits the choice of validation sets for this comparison. Note that an updated version of QD-π, QDπ-2, which overcomes this limitation (see Table 2) and brings other improvements has recently been released,^55^ but the implementation of the method and the ML model used are not yet available. The results are plotted in the Figure 1 and are available in the Supporting Information as a Table S1. Each of the methods discussed here had been trained to reproduce data computed at a different level. Here we test all of them against a reliable benchmark, mostly CCSD(T)/CBS (see Section 2.5), so that we evaluate their absolute accuracy rather than their ability to reproduce the reference level. For comparison, we also include the DFT calculations used in the PM6-ML training data.

In the data sets of small, neutral noncovalent complexes (D442, HB375, R739, and S66), PM6-ML consistently yields root-mean-square errors (RMSE) under 1 kcal/mol, followed by AIQM1, which exhibits a larger error in the D442 data set (RMSE 1.84 kcal/mol). The QD-π method yielded larger errors (RMSE between 2 and 3 kcal/mol) in all data sets except for S66. When charged molecules are considered in the IHB100 data set of ionic hydrogen bonds, PM6-ML provides accurate results with an RMSE of 0.75 kcal/mol, representing a significant improvement over the earlier corrections for PM6. The RMSE yielded by AIQM1 is 2.3 kcal/mol, and this error is not systematic. Conversely, QD-π consistently overestimates the strength of these interactions, resulting in an RMSE of up to 7.48 kcal/mol. Additional benchmarks of QD-π and AIQM1 in small model systems, and their comparison to a broader selection of SQM methods, can also be found in ref (59), and QD-π performs rather well there.

In the next two sets of larger noncovalent complexes, L7 and S12L, PM6-ML yields larger errors (RMSE 2.47 and 6.36 kcal/mol, respectively), but these are not significantly different from the errors of the DFT calculations (0.92 and 5.98 kcal/mol) that this method has been trained to reproduce. It is also noteworthy that the interaction energies in this data set are an order of magnitude larger than in the smaller systems. Both AIQM1 and QD-π are unable to accurately describe the interaction energies in the larger systems, with errors of 19.5 and 62.4 kcal/mol in the S12L data set.

This brief analysis demonstrates that, in addition to their limited coverage of chemical space, both AIQM1 and QD-π are substantially less accurate than PM6-ML and are not transferable to larger molecular systems.

### 3.3 Validation: Noncovalent Interactions

Due to their importance in all large molecular systems, noncovalent interactions are a key target of the PM6-ML method. They are well represented in the training set, and PM6-ML is expected to provide significant improvement over the empirical corrections previously used with SQM methods.

#### 3.3.1 Small Noncovalent Complexes

**Figure 2:**
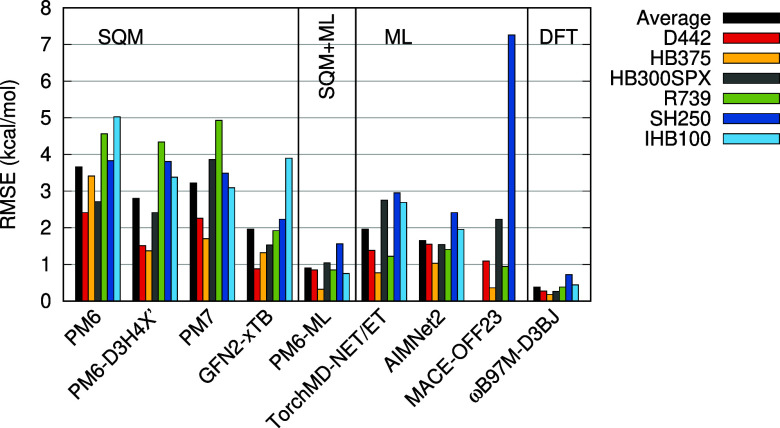
Errors of the tested methods in the validation subset of the Noncovalent Interactions Atlas data sets. Root mean square error of interaction energies in kcal/mol. MACE-OFF23 is not applicable to the IHB100 data set.

First, we analyze noncovalent interactions in smaller complexes, similar in size to the systems used in the training. Here, we take advantage of the NCIAtlas database, which comprises data sets representing separate classes of noncovalent interactions, providing further insight into the results. Additionally, these data sets feature reference data computed at a true benchmark level, CCSD(T)/CBS. The errors of the tested methods are plotted in Figure 2 and listed in Table S2 in the Supporting Information. The error averaged over the six NCIAtlas data sets is also listed it Table 4.

**Table 4:** Root Mean Square Errors of the Tested Methods, in kcal/mol, Averaged over the Six NCIAtlas Validation Sets, Noncovalent Interactions in Large Systems (L7 and S12l Datasets) and Datasets of Conformation Energies (MPCONF196, SCONF, Amino20 × 4)^a^

method	NCIAtlas	NCI/large	conformers
PM6	3.66	17.94	3.45
PM6-D3H4X′	2.80	**4.47**	4.04
PM7	3.22	11.41	3.37
GFN2-xTB	1.96	**2.71**	2.00
PM6-ML	**0.90**	**4.42**	**0.77**
TorchMD-NET/ET	**1.96**	17.06	**1.05**
AIMNet2	**1.65**	40.57	1.23
MACE-OFF23	2.38^b^	7.34	**0.71**
ωB97M-D3BJ	0.38	3.45	0.40

a Three best results in each column (neglecting the DFT) are highlighted.

b Excluding the IHB100 dataset.

PM6-ML is unquestionably the best-performing method in this test. Not only does it yield the lowest error on average (RMSE averaged over the six data sets is 0.90 kcal/mol), but it also provides the best result in each of them. It is followed by AIMNet2 (avg. RMSE 1.65 kcal/mol) with consistently good results in all the data sets, TorchMD-NET/ET (avg. RMSE 1.96 kcal/mol), and GFN2-xTB (avg. RMSE 1.96 kcal/mol). All the SQM methods have some weak points, most often in the description of σ-hole interactions (SH250 set), repulsive contacts (R739), and ionic hydrogen bonds (IHB100). Among them, GFN2-xTB performs the best, with only the IHB100 set standing out with an error of 3.9 kcal/mol. The standalone ML potentials generally perform better than the SQM methods, with the exception of the very large error of MACE-OFF23 in the SH250 data set (7.3 kcal/mol), which is likely caused by the lack of relevant systems in its training set.

In these validation sets, PM6-ML benefits from having similar systems in the training set. This is also the reason why the TorchMD-NET/ET ML potential, trained on the same data, yields relatively small errors. On the other hand, it is clearly visible in the results that despite this, TorchMD-NET/ET performs worse specifically in the more exotic interactions (σ-hole bonds, hydrogen bonds involving heavier elements), which are represented more sparsely in the training set. The better description of these in PM6-ML indicates that it is the synergy of the SQM physics with the ML correction that enables higher accuracy with the same training data.

However, the analysis of the NCIAtlas data sets demonstrates only the accuracy that can be reached under very favorable conditions, i.e., in systems of similar size to those in the training set. It tests the ability of the models to interpolate the chemical space, but not their ability to extrapolate to larger molecular systems.

#### 3.3.2 Large Noncovalent Complexes

**Figure 3:**
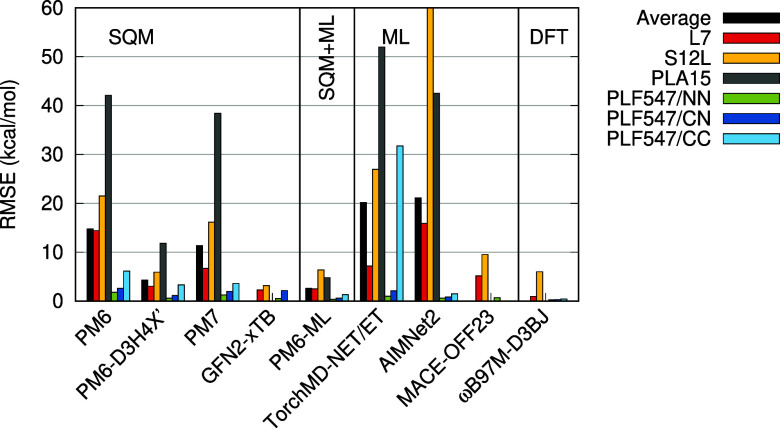
Errors of the tested methods in benchmark data sets of large noncovalent complexes. Root mean square error of interaction energies in kcal/mol. MACE-OFF23 is not applicable to the PLA15 and PLF547 data sets.

Since the intended application of all the studied methods is larger systems, it is necessary to validate their transferability from small model complexes to larger ones where long-range interactions become more important. To do so, we employ several data sets where quality benchmarks [DPLNO-CCSD(T)] are still available. The L7 and S12L are data sets of larger noncovalent complexes with emphasis on π–π stacking. The PLA15 set features even larger models of protein–ligand complexes (15 ligands with surrounding amino acid residues extracted from an experimental structure of the complex). The pairwise interactions in this data set form the PLF547 set, which we include here as well. The results are summarized in Figure 3 and listed in the Supporting Information, Table S3. Additionally, the average of the RMSE in the L7 and S12L sets is reported in Table 4 (PLA15 results are not available for all the methods).

Among the SQM methods, PM6 and PM7 yield very large errors, and the corrections in PM6-D3H4X′ bring them down to an acceptable level, with an average RMSE of 4.5 kcal/mol in the L7 and S12L sets. GFN2-xTB performs excellently here with an RMSE of 2.7 kcal/mol but fails to converge in some of the PLA15 systems.

This test is much more challenging for the ML potentials. Our version of TorchMD-NET/ET, which lacks the description of long-range interactions, yields large errors increasing with the size of the system, up to an RMSE of 52 kcal/mol in the PLA15 set. It is more surprising that the AIMNet2 method does not perform better here, as it includes separate terms for long-range electrostatics and dispersion. Despite this, it exhibits the largest errors, with an RMSE of 65 kcal/mol in the S12L set (where the average interaction energy at the benchmark level is 41 kcal/mol, which translates to a relative error of 160%). Interestingly, it also yields a large error in the PLA15 data set (RMSE of 43 kcal/mol, which amounts to 25% of the average interaction energy in the set), while all the smaller fragments these systems comprise of, in the PLF547 data set, are described much better, with the worst error in the subset of charged–charged species interactions having an RMSE of 1.5 kcal/mol, which translates to only 2.3% of the interaction energies there. Apparently, the model is able to describe smaller systems well but fails to scale to the larger ones. The only pure ML potential that performs better here is the MACE-OFF23 method, which yields an average RMSE of 7.3 kcal/mol in the L7 and S12L sets. It is, however, not applicable to charged systems, so it cannot be tested in the even larger complexes of the PLA15 set. The MACE-OFF23 potential does not include any explicit treatment of long-range interactions, but its training set was extended with a set of larger systems, which is likely the reason why it performs better.

The PM6-ML method works well here, with RMSEs of 2.47, 6.36, and 4.78 kcal/mol in the L7, S12L, and PLA15 sets, respectively. These results should be viewed in the context of the accuracy of the DFT on which the method is trained, which yields RMSEs of 0.92 and 5.98 kcal/mol in the L7 and S12L sets. The excellent result in PLA15 (where the relative error is only 2.9%) clearly demonstrates the transferability of the method to large systems and its ability to handle the strong long-range interactions present there. The advantage of the Δ-ML approach is that these interactions are handled independently at the SQM level, and the ML part of the potential is not forced to extrapolate into a territory not covered by the smaller systems used in training. Further tests in even larger protein–ligand complexes are discussed below.

### 3.4 Validation: Conformations and Torsions

Another quantity important in applications to large molecules is the relative energy of different conformers. This is a well-known weak point of all SQM methods that has not been satisfactorily addressed yet. In larger molecules, the conformation energies result from the interplay of noncovalent interactions, including repulsion defining the steric limits at short-range, with the energetics of the torsions defined by the electronic structure of the molecule. While the former can be addressed by corrections for noncovalent interactions, the description of the torsions is severely limited by the approximations inherent to the SQM methods, and there is no satisfactory solution to it. On the other hand, the latter contribution is localized to only a few atoms around each chemical bond, which makes it a good target for ML potentials.

**Figure 4:**
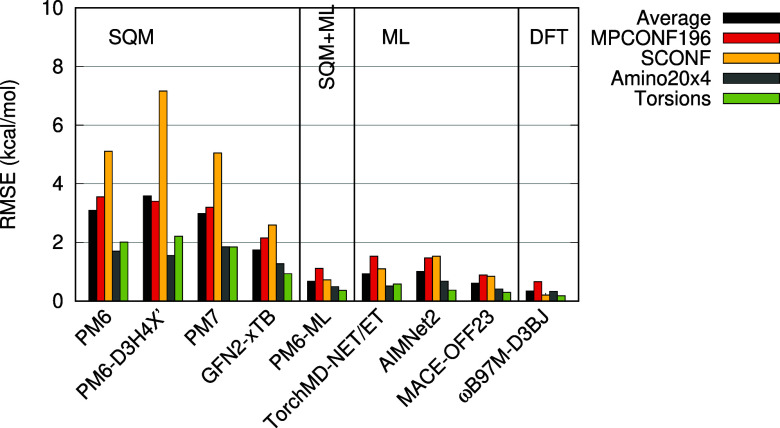
Errors of the tested methods in benchmark data sets of conformation energies and torsional profiles. Root mean square error of the relative energies in kcal/mol.

In this paper, we examine the conformation energies in three benchmark data sets: MPCONF196, which comprises peptides and peptidic macrocycles,^17^ SCONF, which represents sugars,^40^ and Amino20 × 4, which covers selected conformers of the 20 biogenic amino acids.^40^ The results are plotted in Figure 4 and listed in Table S4 in the Supporting Information. It is clear that SQM methods do not yield very good results here. In particular, the sugar conformers in the SCONF data set are very challenging, with the lowest RMSE achieved by GFN2-xTB being as high as 2.59 kcal/mol, which corresponds to 56% in relative terms. Similarly, in the MPCONF196 and Amino20 × 4 sets, GFN2-xTB performs the best among the SQM methods, but even these results are not very good. Its error averaged over the three data sets is 2.00 kcal/mol (see Table 4). It is, however, a significantly better result than that of the next method, PM7, which has an average RMSE of 3.37 kcal/mol.

On the other hand, these benchmarks are relatively easy for all the ML potentials tested. The results are consistently very good, with none of the ML potentials performing worse than the best SQM method. Among the three methods, MACE-OFF23 performs the best, with an RMSE under 0.9 kcal/mol in all the data sets, and an average of 0.71 kcal/mol. Apparently, the conformer energetics are easy to learn, and the training data provide enough information for that. The same applies to the application of ML to correcting the SQM calculation in PM6-ML. It performs consistently well in all three data sets, with an average RMSE of 0.77 kcal/mol. However, even here, PM6-ML benefits from the synergy of SQM and ML, as the standalone TorchMD-NET/ET model does not perform as well, with an average RMSE of 1.05 kcal/mol.

The effect of the energetics of torsions can be isolated using the Torsions data set, which features torsional profiles of 62 bonds in model drug-like molecules.^43^ There, the best SQM result is again obtained with GFN2-xTB, which has an RMSE of 0.93 kcal/mol. The best ML method is MACE-OFF23, with an RMSE of 0.29 kcal/mol, while PM6-ML yields an error of 0.36 kcal/mol. It should be noted that these results already approach the accuracy of the DFT used in the training of these methods, with respect to the CCSD(T) benchmark, which has an RMSE of 0.18 kcal/mol.

**Figure 5:**
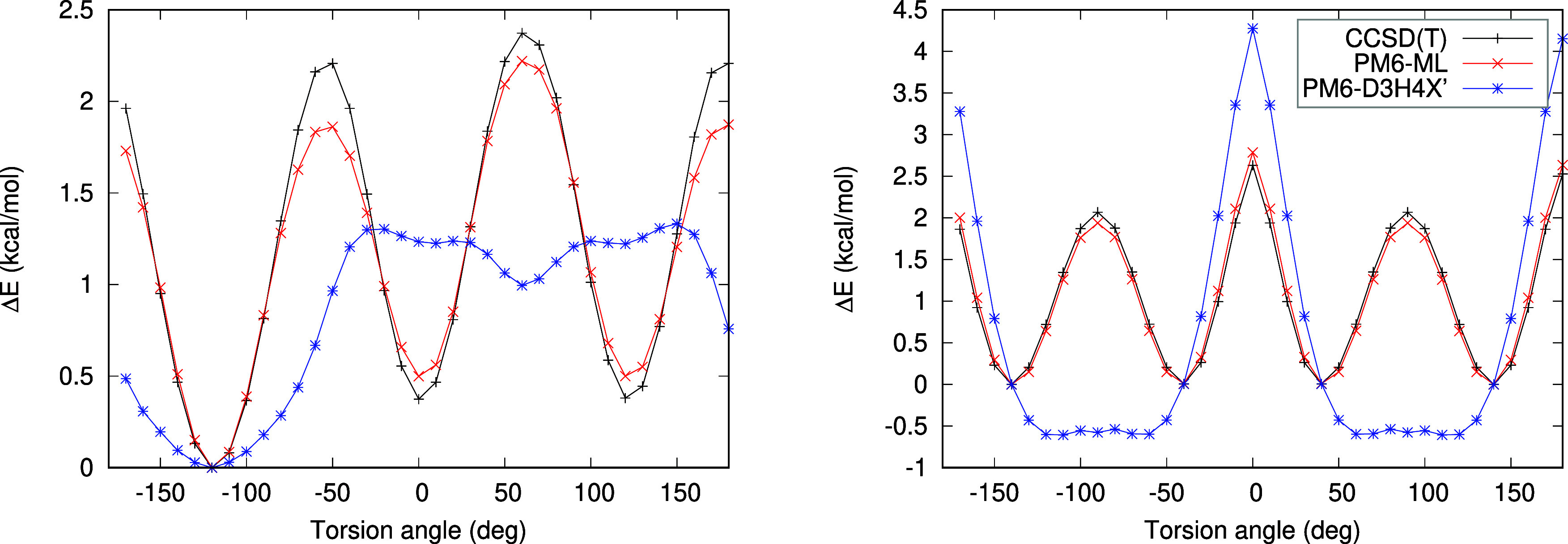
Examples of the torsional plots for 4-methyl-pentene (left) and biphenyl (right) computed with PM6-ML (red) and PM6-D3H4X′ (blue), in comparison to the CCSD(T) benchmark (black).

More importantly, this data set provides additional insight into the phenomena when the torsional profiles are plotted. Two samples from the data set are shown in Figure 5, and all the plots are available in the Supporting Information as Figure S1. It is clear that, in some cases, such as those shown here, PM6-D3H4X′ describes the potential qualitatively incorrectly, with missing barriers and artificial minima. The ML correction in PM6-ML is able to eliminate all these issues, and the resulting potential matches the benchmark almost perfectly. It is also important to note that this excellent result is achieved without reference data systematically sampling torsions in the training set—this information is recovered by the model from randomly sampled conformers only.

### 3.5 Validation with Geometry Optimization, Gradients

The benchmarks presented above are all based on the evaluation of the interaction or conformation energy on a fixed geometry optimized at a higher level. This is the approach commonly used in the field, but it has two important drawbacks. First, the methods have been parametrized on similar geometries, mostly minima obtained at a higher level, which introduces a bias into the result and may lead to omission of errors in other areas of the potential energy surface. Second, such a benchmark is far from practical applications, where it is unlikely that the geometry would be optimized, e.g. using DFT, just to perform a single point SQM calculation on it. On the other hand, the benchmark energies provided in the data sets are never perfectly consistent with the geometries. Usually the geometry has been optimized using DFT-D or MP2, and only the final quantity is calculated at the benchmark level, where the geometry is not an exact minimum. This argument supports benchmarking in fixed geometries.

The best option is to apply both approaches and compare their results. To do this, we have computed the studied quantities (interaction energies and relative energies of the conformers) also in geometries optimized with the tested method. In Table 5 the results of PM6-ML are compared with PM6-D3H4X′. An analogous table of systematic errors (mean signed error, MSE) is available in the Supporting Information as Table S11. In the case of conformation energies, the full MPCONF196 cannot be used because the macrocycles it contains were optimized in a solvent, so we use only the peptides for which the geometries were obtained in the gas phase. This subset is denoted PCONF.

**Table 5:** Error (RMSE in kcal/mol) of PM6-D3H4X′ and PM6-ML in Multiple Validation Datasets Computed in the Fixed, Benchmark Geometries and in Geometries Optimized with the Respective Tested Method

method	PM6-D3H4X′		PM6-ML	
geometries	optimized	fixed	optimized	fixed
D442	1.53	1.51	1.37	0.84
HB375	1.4	1.37	0.38	0.31
HB300SPX	2.38	2.41	1.83	1.04
SH250	3.98	3.88	1.95	1.59
IHB100	17.12	3.38	1.62	0.75
S66	0.83	0.85	0.58	0.4
PCONF	1.35	1.63	0.56	0.67
SCONF	5.79	7.16	0.86	0.73
Amino20 × 4	1.93	1.55	0.46	0.49

In both methods, the results in neutral systems evaluated (it is excluding the IHB100 data set) on the optimized geometries give slightly worse results than in the original ones. In PM6-D3H4X′ there is practically no difference, while in PM6-ML the RMSE error averaged over these data sets increases from 0.76 to 1.01 kcal/mol. Even this can be considered negligible, given the limitation outlined above and the fact that PM6-ML was trained on DFT results but is being benchmarked here against the CCSD(T) reference. Regardless of the changes introduced by the optimization, PM6-ML still consistently and significantly outperforms its predecessor.

The errors in the charged systems, represented by the IHB100 data set of ionic hydrogen bonds, are much larger. Here the PM6-D3H4′ optimization yields geometries where the interaction energies are exaggerated, with a systematic error, MSE, of −2.84 kcal/mol and a RMSE of 17 kcal/mol. PM6-ML behaves much better, the RMSE increases from 0.75 to 1.62 kcal/mol and the MSE remains negligible at 0.12 kcal/mol.

Another way to benchmark the description of the most important part of the potential energy surface is to perform single point calculations in selected nonequilibrium geometries. The NCIAtlas data sets used here provide such scans of the intermolecular distance. The results (provided in the Supporting Information, Table S12) yield similar conclusions to the analysis presented above—PM6-ML describes the nonequilibrium structures with only a small loss of accuracy, while PM6-D3H4X′ exhibits unacceptably large errors in some of the data sets (D442 × 10 and SH250 × 10).

This test indicates that PM6-ML provides a robust description of the geometries of both noncovalent complexes and distinct conformers of the same molecule, and the interaction and conformation energies evaluated on these geometries remain close to the benchmark. A major improvement is that this is also true for the ionic H-bonds, where PM6-D3H4X′ yielded incorrect geometries in which the strength of the interaction was severely overestimated.

To complement the geometry optimizations, we also directly analyze the quality of the PM6-ML gradients. Two data sets are tested: First, we use the validation subset of the five NCIAtlas data sets, again including the nonequilibrium geometries along the dissociation curves of the complexes (2300 geometries in total). Second, we use 100 snapshots from an MD simulation of the 5QD5 inhibitor, which provide a more random sample of nonequilibrium structures accessible at room temperature.

The quality of the PM6-ML gradients, as well as those of PM6 and PM6-D3H4X′ added for comparison, is evaluated against the ωB97M-D3BJ/def2-TZVPPD reference as the root-mean-square difference in each system and averaged over the data set. The results are presented in Table 6. For the NCIAtlas data sets, we also report the errors in the closest and equilibrium geometries separately.

**Table 6:** Error of the Gradients, Evaluated against the ωB97M-D3BJ/def2-TZVPPD Reference as Root Mean Square Difference between the Gradients and Averaged over the Validation Data Sets

data set	gradient error, kcal/mol/Å		
	PM6-ML	PM6	PM6-D3H4X′
5QD5MD snapshots	1.26	12.02	13.23
NCIAtlas all	1.83	11.63	13.65
NCIAtlas closest	3.11	12.70	15.18
NCIAtlas equilibrium	1.73	11.59	13.44

The data show that PM6-ML is able to reproduce the DFT gradients very closely with an average error of less than 2 kcal/mol/Å, which is an order of magnitude better than either PM6 alone or PM6 with the earlier D3H4X′ correction. In the NCIAtlas dissociation curves, larger errors can be observed in the artificial, difficult to describe close contact geometries (obtained by scaling the equilibrium intermolecular distance by 0.8). The accuracy of the gradients is similar to that obtained for the energies, as can be seen from the 5QD5 MD snapshots, where both quantities reach a similar magnitude and the relative error of the gradient (obtained by dividing this gradient difference by the average RMS of the gradient in the set) is 5.9%, close to the error in the relative energies of the sampled conformations, which is 4.6%.

### 3.6 Application to Protein–Ligand Complexes

The final test closely simulates the intended application to large biomolecular systems, specifically the evaluation of protein–ligand interactions. We take advantage of the PL-REX data set we developed earlier,^11^ which features DFT interaction energies in 164 models comprising the ligand and the surrounding part of the protein with ∼1000 atoms. Out of these, we use the 136 systems without zinc ions in this study.

The reference energies were computed using a DFT functional similar to the one used to train PM6-ML, ωB97X-D3BJ, but with a smaller basis set, DZVP-DFT.^38^ Nevertheless, our previous results suggest that this basis set reproduces well the interaction energies obtained with a larger, triple-ζ basis set,^39^ which makes the PL-REX DFT results a suitable reference. Furthermore, we omit the 3-body term in the D3 correction used in PM6-ML because the ωB97X-D3BJ reference data do not include it either. The PM6 calculations use the MOZYME linear scaling algorithm.^13^

**Figure 6:**
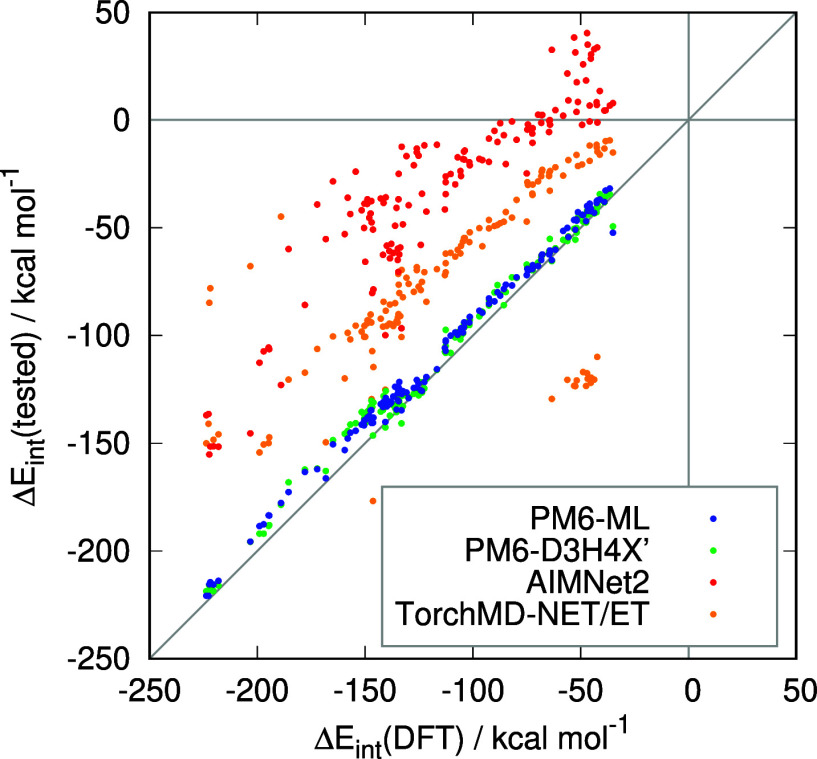
Plot of interaction energies obtained with PM6-ML and other tested methods against the DFT reference. The dots represent the 136 large models of protein–ligand complexes from the PL-REX data set.

We compare PM6-ML with PM6-D3H4X′, which we have already applied successfully to scoring protein–ligand interactions,^11^ and with two ML potentials applicable to the chemical space spanned by the ligands, AIMNet2 and TorchMD-NET/ET (MACE-OFF23 is applicable only to neutral molecules). The key results are summarized in Table 7 and the individual data points are plotted in Figure 6 (the source data are available in the Supporting Information, Table S13).

**Table 7:** Correlation and Error of PM6-ML and Other Tested Methods Compared to the DFT Reference, Evaluated on 136 Large Models of Protein–Ligand Complexes from the PL-REX Dataset^a^

method	*R*^2^	RMSE, kcal/mol	RMSE, relative (%)
PM6-ML	0.994	7.46	6.65
PM6-D3H4X′	0.990	7.65	6.82
AIMNet2	0.822	83.87	74.8
TorchMD-NET/ET	0.362	54.33	48.44

a The relative error is expressed as a percentage of the RMSE with respect to the average magnitude of the interaction energy in the set.

Both PM6-ML and PM6-D3H4X′ reproduce the DFT results with a high degree of accuracy, with an RMSE of approximately 8 kcal/mol (equivalent to a relative error of approximately 7%, given the magnitude of the interaction energies). PM6-ML displays a slight advantage in this regard, however, the difference is inconsequential given that the reference DFT results themselves are subject to a nonnegligible uncertainty due to the utilization of a relatively small basis set. A comparison of these two methods against more accurate benchmark is available in smaller systems of the same kind, namely the PLA15 data set discussed above in Section 3.3. There, PM6-ML is clearly shown to be superior to PM6-D3H4X′. The principal conclusion to be drawn from the results in the PL-REX set is that PM6-ML maintains its high level of performance even when applied to significantly larger systems.

Both ML-only potentials are unable to adequately address this transition. It is unsurprising that the TorchMD-NET/ET potential is ineffective in this context. It is a simplified model that lacks the capacity to describe long-range effects (the cutoff distance defining the atomic environment is only 5 Å), which manifests as a systematic bias toward weaker interaction energies. However, a group of outliers in the other direction is evident in Figure 6, comprising all complexes of the BACE1 protein where the model bears a larger charge than in the other systems. In contrast, the suboptimal performance of AIMNet2 is unexpected. This potential incorporates a physics-based description of long-range interactions (including both electrostatics and dispersion), suggesting that the observed large errors and systematic shift toward more positive interaction energies can be attributed to the parametrization, rather than the form, of the model. It is probable that including terms covering long-range effects is insufficient for developing a scalable model when the training set comprises only smaller systems.

These observations suggest that an ML potential that is scalable to large systems should meet two main requirements: It must have the means to describe long-range interactions, and the associated parameters must be meaningfully determined in training. For the former, the inclusion of physics-based terms such as Coulomb electrostatics and London dispersion should be preferred to increasing the distance cutoff in a free-form ML potential, as the resulting model should be more computationally efficient and easier to train. However, both of these approaches require training data covering large systems, which is a major challenge. They are much more difficult to generate than expanding the coverage of small molecules, and the increasing size of the systems also brings more configurations to be sampled. This is where the Δ-ML approach offers an important advantage—the description of the long-range interactions is decoupled from the ML model and works the same way in systems and configurations not covered by the training data. Since it contains no adjustable parameters, the description of long-range effects above the cutoff distance cannot be worse than in the baseline method. In PM6-ML, this means that the London dispersion is exactly the same as in the DFT-D3 reference, and the electrostatics comes from PM6, whose formalism is sufficient to describe it correctly.

### 3.7 Timing

**Figure 7:**
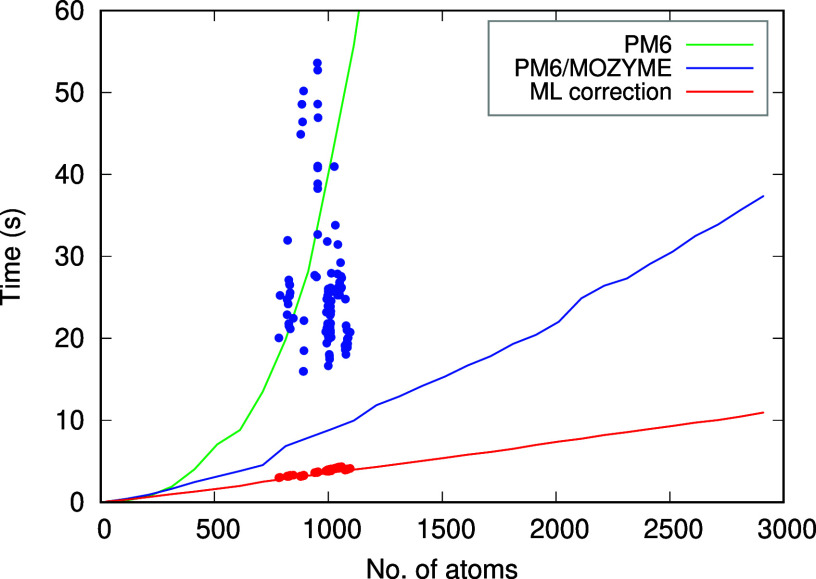
Time required to compute the single point energy of polyalanine chains (lines) and protein–ligand complexes from the PL-REX data set (dots). An additional 11 (out of 136) PL-REX data points with computation times ranging from 60 to 207 s are not shown in the graph.

The timing of PM6-ML calculations is benchmarked using the aforementioned PL-REX protein–ligand complexes and polyalanine α-helices of varying lengths. The PL-REX systems contain between 877 and 1059 atoms and serve as a realistic example of 3D structures with multiple charged sites. The neutral polyalanine chains, which range in length up to 2900 atoms, encompass a more extensive range of system sizes in a systematic manner. Given our focus on PM6 calculations with the MOZYME linear scaling algorithm,^13^ which is not parallelized, all the calculations were conducted on a single CPU core. Although the ML correction can be computed more rapidly with parallelization or on a GPU, this is also the setup we utilize in practice, as it allows for the efficient management of a large number of serial jobs running in parallel. All calculations were performed on a computer with an Intel Xeon Gold 6140 CPU at 2.30 GHz with 96 GB RAM. All MOPAC settings were left at the default values. The results are summarized in a plot in Figure 7.

It is first necessary to distinguish between the canonical and linear-scaling PM6 calculations. PM6 itself scales as *O*(*N*^3^), although the prefactor is smaller than that of more advanced quantum mechanical methods. This makes it possible to perform calculations on larger systems comprising thousands of atoms, although the computational demand remains significant. In practical applications, the use of reduced-scaling algorithms is indispensable. The MOZYME algorithm,^13^ accessible in MOPAC, is an exceptionally efficient solution. In the polyalanine series, the PM6 calculations with MOZYME exhibit nearly linear scaling, with PM6 energy calculations for 1000 atoms completed in 9 s and for 2900 atoms completed in 37 s. The scaling of the ML correction is even more linear, with a computational time of 3.6 s for 100 atoms and 11 s for 2900 atoms. The PL-REX set demonstrates that the chemically and geometrically more complex systems are more demanding for the PM6 calculation, with an average computational time of 32 s. In contrast, the performance of the ML correction is close to that observed in the polyalanine models of similar size (see Figure 7). On average, the ML correction accounts for 13% of the total computational time in these systems.

The above timings are reported for single point energy calculations, a calculation of energy and gradient is slightly more expensive at the PM6/MOZYME level (on average by 9% in the polyalanine helices) and about 2.5 times more expensive for the ML correction (9 s for 1000 atoms), and their timings become comparable. However, in the realistic P–L complexes of similar size, where the PM6 calculations take longer, the ML gradient remains as fast as in the model systems and accounts for about 20% of the total computational time.

The results demonstrate that the ML correction introduces a only a small computational overhead with respect to the underlying SQM calculation. Overall, PM6-ML exhibits excellent performance in systems comprising up to a few thousand atoms and is sufficiently fast for practical applications in more complex computational protocols or large data sets.

## 4 Conclusions

The results presented in this paper show that the PM6-ML method, a Δ-ML approach based on efficient semiempirical QM calculations and a modern ML potential, has unique features that none of these components can currently achieve on their own. Although the accuracy of SQM methods has recently improved, it is now reaching the limits imposed by the fundamental approximations these methods are based on. On the other hand, even the best general ML potentials are limited by the scope of their training data more than methods that include parameter-free physically sound components. This is demonstrated not only by the ML methods’ failure to describe larger molecular systems where long-range interactions play an important role but also by other benchmarks discussed here.

PM6-ML addresses the most severe issue of existing SQM methods, the poor description of conformation energies of flexible molecules, and improves the accuracy of noncovalent interaction energies beyond what was achievable with the empirical corrections used previously. Compared to previous Δ-ML SQM methods, PM6-ML covers a much wider chemical space, enabling its application to real-world chemical problems. In this paper, we demonstrate its applicability to the study of protein–ligand interactions, where it achieves excellent accuracy. It also achieves very good efficiency there, with single-point computations of 1000 atoms taking only about 30 s on a single CPU core.

The implementation of PM6-ML is freely available and includes a direct interface to MOPAC, the most widely used software for SQM calculations. This should facilitate its adoption in all cases where traditional SQM methods have been used.

The PM6-ML method presented here is, however, only the first step in its development. We are already working on extending its training set to include additional chemical elements and to cover other quantities and phenomena beyond those discussed in this paper.

## Data Availability

The trained models of both the PM6-ML correction and the standalone TorchMD-NET/ET potential used in this paper, as well as the wrapper enabling the PM6-ML calculations in MOPAC are available for download from a GitHub repository https://github.com/Honza-R/mopac-ml. The modified MOPAC code with the corresponding interface is available at https://github.com/Honza-R/mopac/tree/pm6-ml. Additionally, the PM6-ML calculations can be also carried out using an unmodified version of MOPAC interfaced to the Cuby framework, as described on the Cuby Web site at http://cuby4.molecular.cz/interface_torchmdnet.html.

## Supporting Information Available

SI_tables_and_figures.pdf—additional tables and figures referenced in the main text, including tables of the errors presented in the paper only as plots (PDF)
SI_validation_outputs.zip—data set calculation outputs for all the benchmarked methods and validation sets, which contain individual results as well as additional statistical measures (ZIP)
