# Supplementary material for: PM6-ML: The Synergy of Semiempirical Quantum Chemistry and Machine Learning Transformed into a Practical Computational Method
Source: J Chem Theory Comput. 2025 Jan 3;21(2):678–90. doi: 10.1021/acs.jctc.4c01330 (PMC11780751; doi:10.1021/acs.jctc.4c01330)
Supplement: Supplementary file 1 — ct4c01330_si_001.pdf [file ct4c01330_si_001.pdf]

Supporting information for paper

PM6-ML: The Synergy of Semiempirical Quantum Chemistry and Machine Learning Transformed into a Practical Computational Method

Martin Nováček and Jan Řezáč*

Institute of Organic Chemistry and Biochemistry, Czech Academy of Sciences, 160 00 Prague, Czech Republic

***e-mail: rezac@uochb.cas.cz**

December 4, 2024

1 Tables

Table S1: Comparison of Δ-ML methods and the results obtained at the reference DFT level in the validation subsets covering H, C, N and O elements. RMSE in kcal/mol. These data are plotted in the main text in Fig. ??.

Dataset	PM6-ML	AIQM1	QD-π	ωB97M-D3BJ
D442	0.46	1.84	3.11	0.23
HB375	0.32	1.02	2.19	0.18
R739	0.98	0.95	2.49	0.37
IHB100	0.75	2.31	7.48	0.44
S66	0.37	0.96	1.33	0.26
L7	2.47	6.40	38.77	0.92
S12L	6.36	19.46	62.44	5.98
MPCONF196	1.11	1.30	2.77	0.66
SCONF	0.72	1.05	3.31	0.20
Torsions	0.37	0.31	0.91	0.17
Avg.	1.39	3.56	12.48	0.94

Table S2: Errors (RMSE in kcal/mol) of the tested methods in the validation subset of the NCIAtlas datasets of benchmark interaction energies. These data are plotted in the main text in Fig. ??.
[a] The average excludes the IHB100 set, MACE-OFF23 is not applicable to charged systems.

Method	D442	HB375	HB300SPX	R739	SH250	IHB100	Avg.
PM6	2.41	3.41	2.71	4.56	3.83	5.03	3.66
PM6-D3H4X’	1.51	1.37	2.41	4.34	3.81	3.38	2.80
PM7	2.26	1.70	3.86	4.93	3.49	3.09	3.22
GFN2-xTB	0.88	1.32	1.53	1.92	2.23	3.90	1.96
PM6-ML	0.85	0.32	1.04	0.85	1.56	0.75	0.90
TorchMD-NET/ET	1.38	0.77	2.75	1.22	2.95	2.69	1.96
AIMNet2	1.55	1.03	1.54	1.41	2.41	1.95	1.65
MACE-OFF23	1.09	0.36	2.23	0.94	7.26	N/A	2.38[a]
ωB97M-D3BJ	0.27	0.18	0.26	0.38	0.72	0.44	0.38

Table S3: Errors (RMSE in kcal/mol) of the tested methods in the datasets of large non-covalent complexes. The fragments of the PLA15 dataset form the PLF547 set, which is separated here into the interactions on neutral-neutral (NN), charged-neutral (CN) and charged-charged(CC) molecular pairs. These data are plotted in the main text in Fig. ??.

[a] MACE-OFF23 is not applicable to charged systems.

[b] GFN2-xTB failed to converge in some of the PLA15 and PLF547 complexes.

[c] DFT results are not available for the PLA15 set.

Method	L7	S12L	PLA15	PLF547/NN	PLF547/CN	PLF547/CC	Avg.
PM6	14.39	21.49	42.06	1.79	2.61	6.12	14.74
PM6-D3H4X'	3.02	5.91	11.82	0.56	1.11	3.30	4.29
PM7	6.67	16.14	38.40	1.24	1.99	3.59	11.34
GFN2-xTB	2.26	3.16	N/A[b]	0.50	2.13	N/A[b]	N/A
PM6-ML	2.47	6.36	4.78	0.37	0.58	1.31	2.64
TorchMD-NET/ET	7.17	26.94	51.99	0.97	2.09	31.74	20.15
AIMNet2	15.86	65.27	42.54	0.58	0.83	1.47	21.09
MACE-OFF23	5.16	9.51	N/A[a]	0.67	N/A[a]	N/A[a]	N/A
ωB97M-D3BJ	0.92	5.98	N/A[c]	0.24	0.28	0.39	N/A

Table S4: Errors (RMSE in kcal/mol) of the tested methods in the datasets of conformation energies and torsional profiles. These data are plotted in the main text in Fig. ??.

Method	MPCONF196	SCONF	Amino20x4	Torsions	Avg.
PM6	3.55	5.11	1.70	2.01	3.09
PM6-D3H4X'	3.40	7.16	1.55	2.21	3.58
PM7	3.20	5.05	1.85	1.84	2.98
GFN2-xTB	2.15	2.59	1.27	0.93	1.74
PM6-ML	1.11	0.72	0.49	0.36	0.67
TorchMD-NET/ET	1.53	1.10	0.51	0.58	0.93
AIMNet2	1.47	1.53	0.68	0.37	1.01
MACE-OFF23	0.89	0.84	0.41	0.29	0.61
ωB97M-D3BJ	0.66	0.20	0.33	0.18	0.34

Table S5: Errors (RMSE in kcal/mol) of all 40 trained PM6-ML models on the SPICE subsets of the training data set.

Seed	Solvated AA	Dipeptides	DES370K	Pubchem data	Ion Pairs
seed1	1.415	0.621	0.366	1.583	3.870
seed2	1.375	0.617	0.363	1.595	3.627
seed3	1.396	0.606	0.369	1.476	3.649
seed4	1.424	0.607	0.366	1.507	3.552
seed5	1.178	0.611	0.359	1.529	3.657
seed6	1.313	0.614	0.354	1.133	3.576
seed7	1.474	0.630	0.368	1.540	3.725
seed8	1.126	0.598	0.350	1.586	3.577
seed9	1.518	0.644	0.400	1.891	4.147
seed10	1.417	0.647	0.378	1.834	3.735
seed11	1.341	0.617	0.370	1.500	3.918
seed12	1.162	0.624	0.354	1.205	3.665
seed13	1.251	0.625	0.365	1.547	3.707
seed14	1.286	0.619	0.370	1.592	3.804
seed15	1.299	0.617	0.364	1.178	3.783
seed16	2.053	0.854	0.593	2.049	5.043
seed17	1.211	0.617	0.383	1.833	3.644
seed18	1.301	0.631	0.372	1.546	3.673
seed19	1.137	0.603	0.352	1.540	3.601
seed20	1.252	0.616	0.358	1.127	3.694
seed21	1.263	0.612	0.363	1.562	3.828
seed22	1.374	0.648	0.370	1.194	3.552
seed23	1.291	0.628	0.356	1.438	3.687
seed24	1.490	0.627	0.370	1.579	3.635
seed25	1.193	0.616	0.343	1.538	3.588
seed26	1.628	0.778	0.487	1.969	4.514
seed27	1.301	0.639	0.370	1.545	3.621
seed28	1.444	0.631	0.368	1.834	3.722
seed29	1.319	0.622	0.358	1.579	3.704
seed30	1.329	0.622	0.357	1.548	3.709
seed31	1.455	0.706	0.438	1.928	3.947
seed32	1.422	0.623	0.362	1.509	3.707
seed33	1.207	0.589	0.364	1.548	3.563
seed34	1.441	0.640	0.372	1.841	3.789
seed35	1.466	0.631	0.358	1.566	3.627
seed36	1.307	0.589	0.363	1.826	3.595
seed37	1.491	0.621	0.362	1.595	3.647
seed38	1.324	0.647	0.378	1.872	3.701
seed39	1.380	0.635	0.378	1.826	3.847
seed40	1.187	0.629	0.356	1.126	3.655

Table S6: Errors (RMSE in kcal/mol) of all 40 trained PM6-ML models in the NCIAtlas subsets of the training data set.

Seed	D1200	D442x10	HB300SPXx10	HB375x10	Rep739x5	SH250x10	IHB100x10
seed1	0.613	0.496	0.416	0.258	0.814	0.610	0.537
seed2	0.585	0.419	0.391	0.269	0.717	0.541	0.618
seed3	0.526	0.378	0.417	0.274	0.735	0.579	0.527
seed4	0.560	0.429	0.413	0.276	0.719	0.567	0.502
seed5	0.583	0.461	0.390	0.268	0.683	0.597	0.521
seed6	0.520	0.378	0.396	0.291	0.724	0.658	0.552
seed7	0.591	0.444	0.440	0.268	0.755	0.561	0.544
seed8	0.535	0.398	0.426	0.266	0.705	0.539	0.466
seed9	0.664	0.488	0.474	0.307	0.831	0.564	0.585
seed10	0.631	0.462	0.432	0.275	0.707	0.567	0.602
seed11	0.562	0.410	0.397	0.279	0.732	0.616	0.550
seed12	0.589	0.422	0.380	0.253	0.729	0.532	0.550
seed13	0.593	0.457	0.388	0.285	0.760	0.603	0.535
seed14	0.598	0.425	0.433	0.297	0.732	0.654	0.548
seed15	0.581	0.433	0.355	0.277	0.683	0.643	0.562
seed16	1.008	0.776	0.688	0.389	1.334	1.001	0.922
seed17	0.552	0.440	0.433	0.301	0.718	0.603	0.539
seed18	0.613	0.436	0.344	0.238	0.742	0.635	0.586
seed19	0.525	0.371	0.396	0.260	0.700	0.588	0.589
seed20	0.578	0.403	0.401	0.280	0.711	0.581	0.528
seed21	0.522	0.390	0.405	0.262	0.756	0.596	0.588
seed22	0.603	0.443	0.389	0.292	0.718	0.606	0.530
seed23	0.545	0.413	0.391	0.268	0.686	0.586	0.616
seed24	0.577	0.440	0.415	0.278	0.742	0.553	0.564
seed25	0.508	0.379	0.374	0.265	0.737	0.534	0.465
seed26	0.974	0.742	0.619	0.383	1.202	0.759	0.734
seed27	0.593	0.432	0.418	0.261	0.774	0.591	0.518
seed28	0.576	0.434	0.429	0.281	0.713	0.585	0.490
seed29	0.589	0.411	0.399	0.285	0.727	0.570	0.537
seed30	0.588	0.418	0.390	0.272	0.670	0.572	0.535
seed31	0.699	0.564	0.476	0.308	0.903	0.779	0.546
seed32	0.567	0.383	0.433	0.247	0.737	0.617	0.541
seed33	0.524	0.416	0.373	0.241	0.683	0.506	0.610
seed34	0.534	0.386	0.392	0.263	0.709	0.548	0.604
seed35	0.555	0.399	0.386	0.265	0.779	0.583	0.588
seed36	0.621	0.463	0.410	0.288	0.809	0.592	0.564
seed37	0.594	0.414	0.433	0.268	0.768	0.548	0.537
seed38	0.600	0.486	0.451	0.283	0.774	0.553	0.565
seed39	0.583	0.407	0.395	0.320	0.761	0.540	0.471
seed40	0.541	0.412	0.361	0.292	0.696	0.544	0.577

Table S7: Errors (RMSE in kcal/mol) of all 40 trained PM6-ML models in the L7 and S12L datasets, the product of all the RMSEs used as the selection metric, and the total mean-squared-error loss of each model.

Seed	L7	S12L	RMSE product	training loss
seed1	12.377	9.525	2.020	124.970
seed2	82.402	5.002	4.550	122.760
seed3	4.123	11.196	0.390	114.180
seed4	13.998	40.907	5.440	101.040
seed5	8.686	5.470	0.410	112.310
seed6	10.217	67.359	4.750	101.960
seed7	7.962	9.146	1.020	106.530
seed8	2.466	6.356	0.090	108.930
seed9	4.797	29.602	5.630	137.880
seed10	7.657	48.288	7.330	127.020
seed11	8.901	12.656	1.280	114.090
seed12	6.667	22.234	0.860	114.470
seed13	17.395	14.132	2.950	114.400
seed14	10.829	17.883	2.960	123.760
seed15	9.015	3.786	0.270	110.710
seed16	15.572	12.775	551.420	161.350
seed17	3.759	5.870	0.310	117.300
seed18	2.117	31.024	0.690	126.240
seed19	8.681	54.223	3.060	111.600
seed20	20.820	8.405	1.150	105.430
seed21	2.834	10.183	0.280	113.920
seed22	9.637	6.465	0.610	120.390
seed23	20.886	4.002	0.750	108.110
seed24	4.034	9.510	0.520	123.790
seed25	3.886	21.336	0.400	104.150
seed26	10.940	14.256	98.120	153.790
seed27	7.936	5.662	0.510	109.740
seed28	1.781	19.791	0.500	123.310
seed29	20.702	6.994	1.530	115.320
seed30	7.029	7.791	0.500	115.970
seed31	5.287	5.253	2.110	142.050
seed32	4.911	13.727	0.690	116.340
seed33	8.698	37.754	1.940	110.390
seed34	32.708	26.888	10.460	122.950
seed35	18.575	39.558	8.350	128.820
seed36	8.275	16.951	2.370	121.780
seed37	4.562	9.784	0.560	124.850
seed38	5.877	5.298	0.630	134.610
seed39	15.078	5.023	1.020	132.570
seed40	31.421	11.029	1.950	101.660

Table S8: Errors (RMSE in kcal/mol) of all 40 trained TorchMD-NET/ET models on the SPICE subsets of the training data set.

Seed	Solvated Amino Acids	Dipeptides	DES370K	Pubchem data	Ion Pairs
seed1	2.491	1.043	0.694	2.271	34.196
seed2	2.329	0.998	0.886	2.296	32.943
seed3	3.338	2.127	1.165	2.622	34.442
seed4	2.604	1.235	0.761	2.491	34.258
seed5	2.531	1.320	0.967	2.284	33.232
seed6	2.640	1.059	0.728	2.271	33.570
seed7	3.648	2.171	1.001	2.863	36.363
seed8	3.287	1.609	0.818	2.615	34.242
seed9	3.327	1.509	0.915	2.517	33.531
seed10	2.429	1.023	0.656	2.306	33.820
seed11	1.959	1.035	0.642	2.284	33.360
seed12	2.590	1.130	0.681	2.297	33.499
seed13	2.680	1.103	0.708	2.299	33.384
seed14	3.698	1.180	1.173	2.488	32.933
seed15	4.289	1.119	1.063	2.174	32.917
seed16	2.283	1.216	0.683	2.356	34.476
seed17	2.608	1.016	0.664	2.272	33.922
seed18	2.390	1.162	0.723	2.387	33.857
seed19	2.063	0.930	0.583	2.111	34.042
seed20	3.800	1.226	0.838	2.123	32.856
seed21	2.823	1.129	0.685	2.360	33.863
seed22	3.141	1.214	1.132	2.435	35.403
seed23	8.782	1.802	1.188	2.871	33.638
seed24	2.809	1.184	0.700	2.384	33.932
seed25	2.081	0.939	0.656	2.316	33.494
seed26	1.901	0.874	0.613	2.156	33.675
seed27	4.186	3.162	1.683	3.116	32.987
seed28	8.608	1.414	0.909	2.448	34.100
seed29	2.837	1.208	0.719	2.458	34.029
seed30	1.877	0.955	0.703	2.262	33.133
seed31	2.203	1.033	0.666	2.306	34.094
seed32	2.823	1.070	0.785	2.308	34.827
seed33	2.417	1.161	0.673	2.319	34.477
seed34	2.474	1.363	0.756	2.515	33.902
seed35	1.938	1.188	0.689	2.343	34.696
seed36	3.379	1.513	0.775	2.511	35.000
seed37	2.506	1.261	0.814	2.564	34.311
seed38	2.472	1.509	0.809	2.575	34.791
seed39	3.602	1.869	0.814	2.618	34.354
seed40	3.366	1.078	0.894	2.326	33.223

Table S9: Errors (RMSE in kcal/mol) of all 40 trained TorchMD-NET/ET models on the NCIAtlas subsets of the training data set.

Seed	D1200	D442x10	HB300SPXx10	HB375x10	Rep739x5	SH250x10	IHB100x10
seed1	0.655	0.523	0.497	0.372	0.901	0.657	0.712
seed2	0.650	0.496	0.472	0.357	0.898	0.599	0.864
seed3	0.804	0.746	0.911	0.546	1.120	1.077	1.907
seed4	0.833	0.658	0.574	0.431	1.134	0.855	1.073
seed5	0.743	0.655	0.627	0.361	1.033	0.698	1.130
seed6	0.708	0.527	0.523	0.409	0.851	0.612	0.909
seed7	1.225	1.002	0.912	0.602	1.663	1.723	1.550
seed8	0.877	0.774	0.719	0.529	1.265	1.066	1.512
seed9	0.994	0.806	0.658	0.531	0.928	0.996	1.066
seed10	0.627	0.463	0.481	0.404	0.931	0.682	0.723
seed11	0.652	0.540	0.480	0.365	0.964	0.674	0.692
seed12	0.738	0.582	0.508	0.426	0.911	0.726	0.811
seed13	0.643	0.472	0.538	0.447	0.995	0.740	0.759
seed14	0.856	0.771	0.669	0.480	0.910	0.949	1.743
seed15	0.784	0.666	0.755	0.488	1.025	0.919	1.428
seed16	0.716	0.564	0.563	0.437	1.007	0.753	0.728
seed17	0.674	0.522	0.531	0.422	0.942	0.797	0.706
seed18	0.783	0.632	0.532	0.406	1.114	0.652	0.813
seed19	0.568	0.452	0.419	0.340	0.740	0.564	0.665
seed20	0.732	0.628	0.781	0.509	1.141	0.936	1.079
seed21	0.758	0.619	0.547	0.416	1.051	0.920	0.849
seed22	0.985	0.827	0.718	0.475	0.917	1.043	1.950
seed23	1.161	1.084	0.776	0.592	1.037	1.145	2.038
seed24	0.789	0.591	0.645	0.449	1.021	0.784	0.883
seed25	0.645	0.521	0.469	0.395	1.031	0.744	0.602
seed26	0.601	0.503	0.408	0.381	0.836	0.582	0.693
seed27	1.582	1.551	1.342	0.717	1.453	2.049	2.742
seed28	0.837	0.726	0.811	0.395	0.872	1.257	1.486
seed29	0.636	0.563	0.567	0.428	1.109	0.784	0.809
seed30	0.678	0.508	0.490	0.350	0.874	0.674	0.600
seed31	0.658	0.585	0.487	0.389	1.053	0.672	0.698
seed32	0.854	0.772	0.589	0.472	0.847	0.804	1.247
seed33	0.723	0.570	0.494	0.427	1.030	0.781	0.774
seed34	0.836	0.697	0.583	0.448	1.143	0.933	0.963
seed35	0.651	0.490	0.527	0.383	0.930	0.683	0.871
seed36	0.787	0.676	0.650	0.470	1.207	0.957	1.003
seed37	0.892	0.832	0.641	0.557	0.915	0.935	1.134
seed38	0.861	0.773	0.733	0.489	1.202	1.167	1.042
seed39	0.862	0.735	0.680	0.601	1.263	1.085	1.283
seed40	0.708	0.577	0.589	0.418	1.015	0.957	0.997

Table S10: Errors (RMSE in kcal/mol) of all 40 trained TorchMD-NET/ET models in the L7 and S12L datasets, the product of all the RMSEs used as the selection metric, and the total mean-squared-error loss of each model.

Seed	L7	S12L	RMSE product	training loss
seed1	10.894	46.116	1878	179.590
seed2	10.214	21.861	879	171.220
seed3	15.368	32.693	257981	189.580
seed4	13.595	60.859	24415	195.060
seed5	35.390	44.419	34637	179.130
seed6	37.878	59.723	13266	172.500
seed7	9.099	25.336	569850	235.800
seed8	20.885	19.761	84041	207.560
seed9	14.760	37.457	59139	193.350
seed10	51.767	45.092	7695	183.990
seed11	51.970	95.448	13666	179.110
seed12	18.926	57.758	8352	184.880
seed13	15.239	28.930	2894	186.650
seed14	23.238	73.930	229709	189.060
seed15	11.359	54.135	58147	186.210
seed16	13.280	29.867	3348	191.050
seed17	12.820	27.039	1964	182.490
seed18	11.877	22.407	2725	192.690
seed19	17.823	77.441	1126	163.280
seed20	11.410	137.795	90319	177.740
seed21	11.058	69.231	11718	187.570
seed22	11.891	106.556	244419	185.400
seed23	19.161	38.483	1873250	191.470
seed24	19.339	65.340	22769	194.250
seed25	12.117	24.247	840	184.520
seed26	12.532	160.034	2349	170.850
seed27	16.122	41.206	29289999	188.820
seed28	11.563	52.556	178233	195.080
seed29	27.222	52.152	17863	194.450
seed30	14.429	50.938	1449	174.020
seed31	15.323	20.683	1361	178.670
seed32	22.683	90.597	60902	184.630
seed33	23.786	26.830	5203	182.950
seed34	13.913	33.301	15745	198.120
seed35	30.175	143.077	19825	185.230
seed36	31.802	77.851	162574	197.060
seed37	15.790	59.491	54697	177.050
seed38	13.007	26.227	32140	206.070
seed39	12.319	26.750	73917	204.390
seed40	10.841	82.080	21751	181.490

Table S11: Systematic error (MSE in kcal/mol) of PM6-D3H4X' and PM6-ML in multiple validation datasets computed in the fixed, benchmark geometries and in geometries optimized with the respective tested method.

Method	PM6-D3H4X'		PM6-ML	
Geometries	Optimized	Fixed	Optimized	Fixed
D442	-0.21	0.63	-0.52	0.09
HB375	-0.42	0.23	0.02	0.13
HB300SPX	-0.48	1.12	-0.89	-0.17
SH250	-0.90	1.34	0.47	0.84
IHB100	-2.84	2.15	0.12	0.29
S66	0.06	0.37	-0.28	-0.10
PCONF	0.00	0.00	0.00	0.00
SCONF	-0.70	-0.54	-0.66	-0.55
Amino20x4	-0.73	-0.62	0.05	0.08

Table S12: Error (RMSE in kcal/mol) of PM6-D3H4X' and PM6-ML in the NCIAtlas validation datasets computed for equilibrium geometries only and for the intermolecular dissociation curves.

Method	PM6-D3H4X'		PM6-ML	
	Equilibrium	Curves	Equilibrium	Curves
D442×10	1.51	2.92	0.85	1.30
HB375×10	1.37	1.71	0.32	0.45
HB300SPX×10	2.41	2.62	1.04	1.04
SH250×10	3.81	10.26	1.56	2.00
IHB100×10	3.38	3.52	0.75	0.83
R739×5	4.34	3.75	0.85	0.57

Table S13: Interaction energies (in kcal/mol) in the protein-ligand complexes from the PL-REX dataset. The DFT calculations were performed at ωB97X-D3BJ/DZVP-DFT level. These data were used to generate the plot in Fig. ?? in the main text of the paper.

Protein	Ligand	DFT	AIMNet2	PM6-D3H4'	PM6-ML	TorchMDNet/ET
002-HIV-PR	1HSG	-159.436	-52.908	-145.606	-153.206	-119.954
002-HIV-PR	1HXW	-164.946	-28.441	-148.582	-150.509	-100.370
002-HIV-PR	1IZH	-185.506	-59.876	-168.181	-172.663	-120.461
002-HIV-PR	1T3R	-142.718	-36.419	-135.344	-131.833	-92.185
002-HIV-PR	2Q54	-148.952	-36.715	-135.345	-137.978	-92.945
002-HIV-PR	2Q55	-158.114	-36.009	-144.296	-147.773	-98.713
002-HIV-PR	2Q5K	-154.373	-23.932	-140.720	-144.195	-95.394
002-HIV-PR	3EKX	-147.017	-47.477	-132.569	-140.567	-129.535
002-HIV-PR	3EL1	-172.163	-39.170	-161.734	-162.006	-106.202
002-HIV-PR	3NU3	-134.550	-36.781	-127.566	-124.091	-90.108
002-HIV-PR	3OXC	-168.252	-55.232	-162.901	-166.300	-149.527
002-HIV-PR	5HVP	-177.938	-85.796	-162.260	-163.345	-117.252
002-HIV-PR	model13a	-156.858	-43.564	-141.337	-145.112	-101.785
002-HIV-PR	model13c	-149.031	-38.848	-136.047	-138.501	-94.295
002-HIV-PR	model13d	-150.288	-38.787	-136.914	-139.279	-95.522
002-HIV-PR	model16a	-148.081	-45.384	-134.490	-137.411	-93.325
002-HIV-PR	model16e	-141.468	-38.439	-128.109	-130.790	-84.299
002-HIV-PR	model16f	-150.523	-50.067	-138.703	-141.745	-100.585
002-HIV-PR	model22a	-147.097	-37.616	-130.214	-134.578	-93.965
002-HIV-PR	model22d	-151.568	-41.802	-135.538	-141.379	-98.222
002-HIV-PR	model23a	-140.381	-35.816	-125.736	-128.990	-88.542
002-HIV-PR	model25d	-147.422	-43.323	-132.996	-134.831	-90.290
003-CK2	1F0Q	-41.139	13.467	-34.357	-36.871	-16.049
003-CK2	1J91	-38.947	4.435	-38.192	-37.964	-13.193
003-CK2	1M2P	-45.809	-0.652	-41.487	-38.800	-18.952
003-CK2	1M2Q	-49.296	-2.175	-44.180	-43.982	-20.620
003-CK2	1M2R	-51.230	8.383	-45.191	-42.754	-21.361
003-CK2	1ZOE	-45.791	6.781	-42.108	-40.251	-11.840
003-CK2	1ZOG	-42.556	8.352	-42.734	-38.468	-11.420
003-CK2	1ZOH	-51.919	3.775	-50.067	-46.093	-16.180
003-CK2	2OXD	-38.405	4.565	-35.488	-32.658	-9.750
003-CK2	2OXX	-42.194	-1.131	-39.209	-37.276	-14.686
003-CK2	2OXY	-36.497	6.793	-34.482	-31.791	-9.406
003-CK2	3KXG	-42.526	7.039	-40.485	-37.624	-12.598
003-CK2	3KXH	-134.643	-70.487	-127.746	-125.436	-71.414
003-CK2	3KXM	-58.057	2.007	-55.585	-51.467	-25.787

003-CK2	3KXN	-35.022	7.829	-49.309	-52.297	-15.051
003-CK2	3PVG	-75.017	-24.707	-67.078	-71.915	-47.102
004-AR	1US0	-116.681	-11.483	-115.973	-115.624	-69.220
004-AR	2IKG	-135.387	-40.717	-135.541	-134.388	-93.399
004-AR	2IKH	-121.544	-42.521	-120.043	-119.304	-84.304
004-AR	2IKI	-122.467	-11.745	-124.526	-121.535	-70.612
004-AR	2IKJ	-125.206	-16.494	-127.796	-122.317	-77.360
004-AR	4LAU	-123.618	-34.156	-126.492	-125.726	-75.075
004-AR	4LAZ	-129.806	-39.535	-128.485	-129.072	-75.984
004-AR	4LB3	-130.783	-16.731	-127.905	-126.488	-73.191
004-AR	4LB4	-126.052	-14.992	-124.316	-120.630	-68.252
004-AR	4LBR	-133.005	-12.401	-128.024	-127.216	-69.541
004-AR	4LBS	-126.029	-13.200	-125.856	-124.201	-69.641
004-AR	4QXI	-134.458	-24.944	-131.580	-130.506	-76.369
004-AR	4XZH	-127.370	-20.924	-127.028	-124.326	-79.749
004-AR	4XZI	-124.183	-57.893	-121.704	-125.424	-78.881
005-Cath-D	6QBG	-141.732	-62.566	-134.596	-133.247	-95.899
005-Cath-D	6QBH	-134.751	-59.676	-129.236	-130.088	-91.594
005-Cath-D	6QCB	-138.797	-60.820	-131.137	-130.758	-94.548
005-Cath-D	model08	-134.845	-64.891	-127.602	-126.942	-93.973
005-Cath-D	model22	-139.915	-58.279	-131.283	-132.929	-95.545
005-Cath-D	model24	-133.666	-59.112	-126.172	-125.475	-90.949
005-Cath-D	model25	-149.974	-65.760	-140.743	-139.309	-100.634
005-Cath-D	model31	-136.938	-61.710	-132.086	-128.297	-95.584
005-Cath-D	model35	-137.815	-57.470	-131.911	-131.093	-94.792
005-Cath-D	model36	-112.987	-41.494	-107.532	-105.787	-71.956
006-BACE1	5QCO	-52.470	31.426	-55.486	-50.864	-123.492
006-BACE1	5QCP	-53.010	38.355	-50.423	-46.325	-123.308
006-BACE1	5QCR	-132.453	-48.354	-132.806	-125.854	-82.185
006-BACE1	5QCT	-138.476	-64.205	-137.214	-131.784	-85.672
006-BACE1	5QCU	-134.121	-62.389	-130.646	-121.558	-80.315
006-BACE1	5QCV	-43.669	32.870	-43.304	-43.037	-120.463
006-BACE1	5QCX	-47.485	18.404	-47.528	-46.907	-123.388
006-BACE1	5QCY	-63.325	32.608	-65.504	-64.995	-129.422
006-BACE1	5QCZ	-42.338	33.697	-40.347	-37.646	-109.966
006-BACE1	5QD0	-51.837	17.597	-52.200	-46.683	-120.791
006-BACE1	5QD1	-45.141	30.513	-43.119	-41.275	-119.553
006-BACE1	5QD2	-47.054	40.353	-42.111	-39.911	-117.470
006-BACE1	5QD3	-45.228	28.599	-43.347	-42.271	-121.926
006-BACE1	5QD5	-48.822	25.904	-47.107	-44.043	-116.983
006-BACE1	5QD9	-56.152	21.624	-56.068	-50.026	-120.561

006-BACE1	5QDA	-46.774	35.072	-43.872	-42.195	-120.115
007-JAK1	4E4L	-85.629	-6.974	-85.914	-78.440	-47.211
007-JAK1	4E4N	-90.019	-5.016	-80.039	-84.183	-49.118
007-JAK1	4E5W	-112.641	-34.396	-104.835	-107.161	-62.005
007-JAK1	4EHZ	-146.744	-80.303	-133.218	-140.028	-107.299
007-JAK1	4EI4	-81.834	-0.648	-73.011	-76.783	-45.221
007-JAK1	4FK6	-104.602	-18.067	-98.993	-98.615	-56.313
007-JAK1	4I5C	-88.563	-10.162	-76.584	-79.923	-48.707
007-JAK1	4IVB	-87.282	-1.434	-81.006	-81.448	-47.818
007-JAK1	4IVC	-104.103	-14.596	-94.906	-96.184	-54.772
007-JAK1	4IVD	-107.100	-17.300	-98.473	-99.639	-60.267
007-JAK1	4K6Z	-92.596	-8.557	-83.597	-82.771	-46.383
007-JAK1	4K77	-64.250	-0.019	-55.103	-60.522	-32.795
008-Trypsin	1K1I	-199.042	-112.648	-191.948	-188.483	-154.244
008-Trypsin	1K1J	-197.068	-107.311	-191.953	-187.638	-150.600
008-Trypsin	1K1L	-194.468	-106.313	-188.027	-183.531	-147.233
008-Trypsin	1K1M	-194.773	-105.602	-188.420	-183.460	-149.937
008-Trypsin	1K1N	-146.330	-50.772	-146.410	-140.537	-176.804
008-Trypsin	2ZHD	-223.671	-136.988	-218.661	-220.848	-150.025
008-Trypsin	2ZQ2	-222.638	-136.460	-219.154	-220.769	-140.998
008-Trypsin	3LJJ	-220.327	-151.431	-218.609	-215.641	-148.409
008-Trypsin	3LJO	-218.065	-151.615	-216.112	-213.894	-145.918
008-Trypsin	5MNG	-140.649	-99.883	-142.626	-140.138	-125.143
008-Trypsin	5MO2	-133.110	-96.639	-140.799	-134.676	-100.651
008-Trypsin	6SY3	-188.975	-122.924	-178.598	-177.715	-44.813
008-Trypsin	6T0M	-221.793	-151.554	-216.735	-214.565	-78.089
008-Trypsin	6T0P	-222.309	-155.224	-216.015	-215.558	-84.766
008-Trypsin	6T5W	-203.253	-145.425	-195.736	-195.682	-67.766
009-CDK2	3QQK	-61.680	4.623	-59.479	-60.144	-21.901
009-CDK2	3QTQ	-72.420	-6.250	-68.782	-69.209	-34.048
009-CDK2	3QTR	-68.080	2.336	-62.803	-62.834	-23.886
009-CDK2	3QTS	-74.760	-4.410	-68.296	-69.037	-28.846
009-CDK2	3QTU	-136.080	-49.087	-133.760	-123.822	-84.951
009-CDK2	3QTW	-79.690	-9.157	-73.272	-72.994	-37.056
009-CDK2	3QTX	-110.260	-25.028	-108.102	-100.085	-61.570
009-CDK2	3QTZ	-101.400	-20.983	-92.611	-91.343	-52.062
009-CDK2	3QU0	-108.200	-29.817	-100.878	-98.589	-63.340
009-CDK2	3QXP	-112.610	-26.160	-108.080	-102.829	-66.396
009-CDK2	3R8U	-69.770	2.517	-66.093	-67.692	-28.476
009-CDK2	3R8V	-64.550	-0.311	-60.826	-62.322	-24.512
009-CDK2	3R8Z	-55.730	9.144	-54.056	-54.271	-18.549

009-CDK2	3R9D	-112.600	-28.856	-97.324	-102.227	-70.678
009-CDK2	3R9N	-68.240	-1.321	-63.576	-64.892	-26.038
009-CDK2	3RAH	-74.470	-2.053	-68.141	-69.915	-30.066
009-CDK2	3RAK	-97.000	-18.018	-91.045	-88.480	-50.108
009-CDK2	3RAL	-105.740	-23.616	-101.790	-97.952	-61.873
009-CDK2	3RJC	-67.770	2.433	-62.496	-62.304	-23.115
009-CDK2	3RK5	-146.140	-78.654	-131.067	-138.156	-114.623
009-CDK2	3RK7	-92.550	-19.343	-85.972	-85.236	-50.277
009-CDK2	3RK9	-72.320	-10.518	-69.723	-68.675	-34.768
009-CDK2	3RKB	-72.210	-3.800	-68.498	-67.332	-29.918
009-CDK2	3RMF	-103.770	-14.110	-95.181	-94.246	-54.714
009-CDK2	3RNI	-95.570	-18.511	-88.956	-89.442	-58.564
009-CDK2	3RPV	-105.500	-25.925	-100.025	-96.642	-58.341
009-CDK2	3RPY	-84.740	-20.428	-79.802	-76.377	-42.707
009-CDK2	3S00	-64.690	-5.506	-61.660	-62.122	-25.960
009-CDK2	3S0O	-64.210	-2.092	-61.311	-61.278	-23.659
009-CDK2	3S1H	-103.980	-18.245	-96.233	-93.811	-54.826
009-CDK2	3SQQ	-101.630	-19.616	-95.178	-93.943	-56.646

2 Torsional profile plots

Figure S1: Plots of torsional profiles from the Torsions dataset. PM6-ML (red) and PM6-D3H4X' (blue) compared to the CCSD(T) reference (black). These are single-point calculations on relaxed torsional profiles - the discontinuities in the profile correspond to change of other coordinate than the one being scanned.

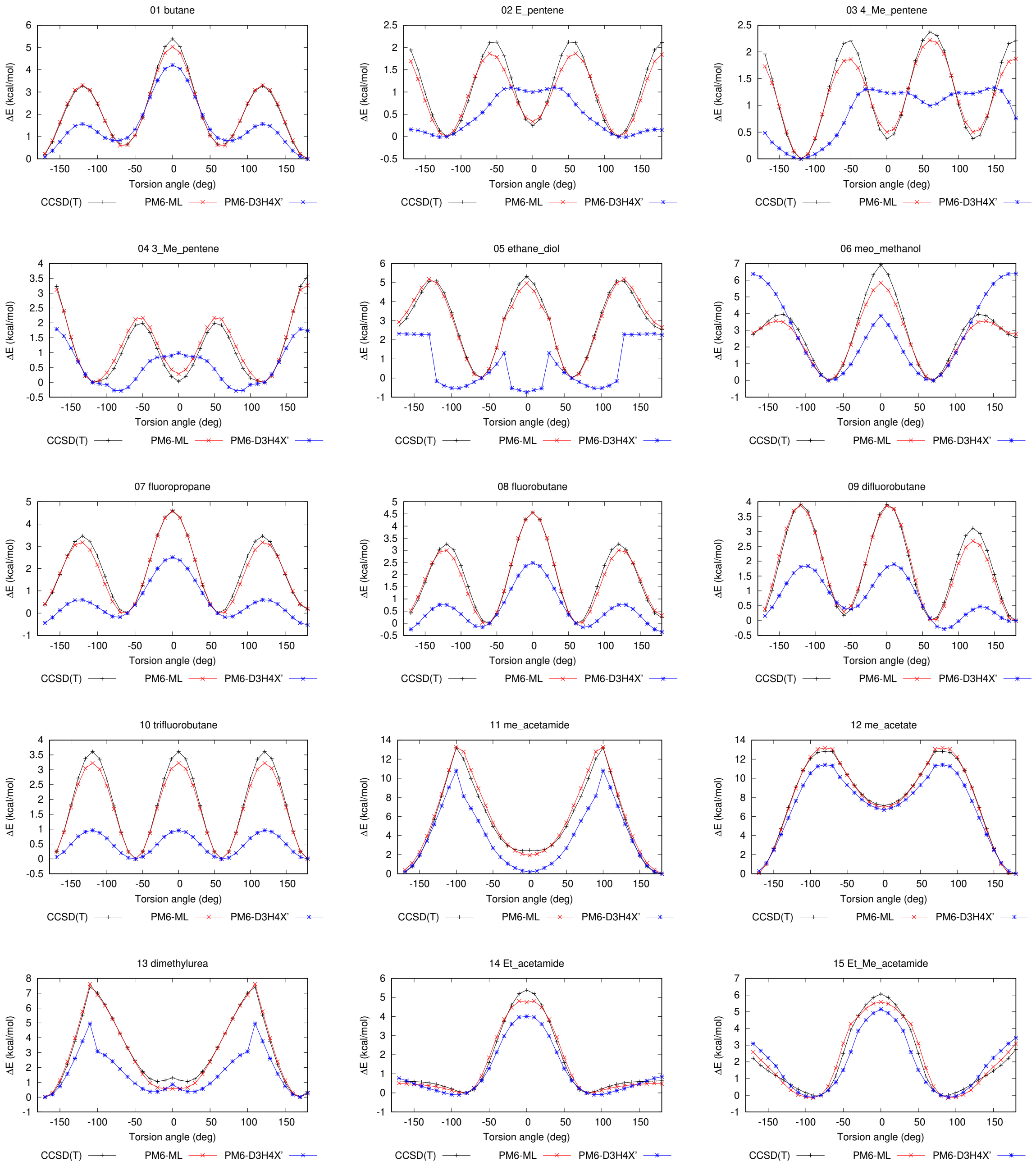

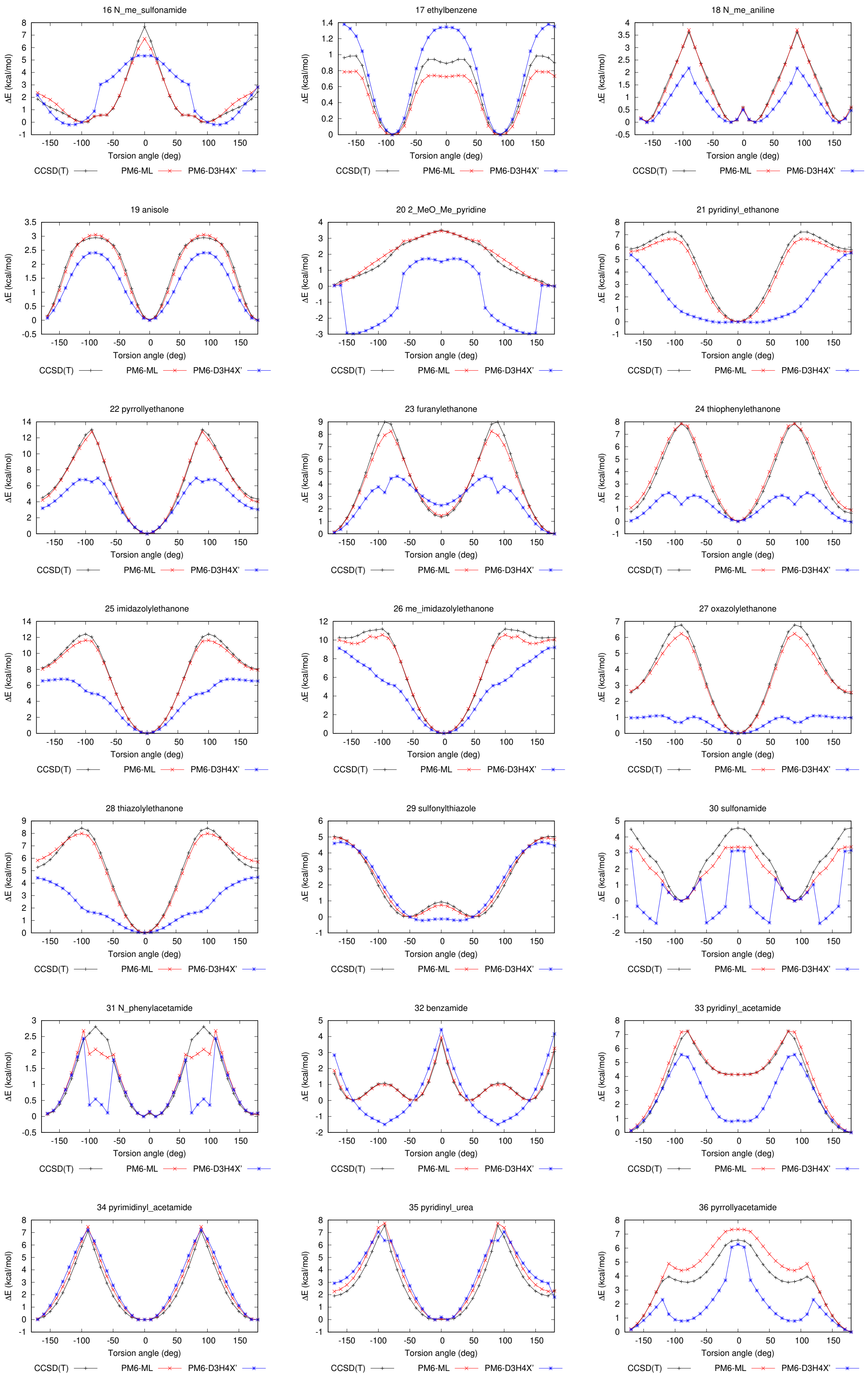
16 N_me_sulfonamide
17 ethylbenzene
18 N_me_aniline
19 anisole
20 2_MeO_Me_pyridine
21 pyridinyl_ethanone
22 pyrrollyethanone
23 furanylethanone
24 thiophenylethanone
25 imidazolylethanone
26 me_imidazolylethanone
27 oxazolylethanone
28 thiazolylethanone
29 sulfonylthiazole
30 sulfonamide
31 N_phenylacetamide
32 benzamide
33 pyridinyl_acetamide
34 pyrimidinyl_acetamide
35 pyridinyl_urea
36 pyrrollyacetamide
ΔE (kcal/mol)
Torsion angle (deg)
CCSD(T)
PM6-ML
PM6-D3H4X'

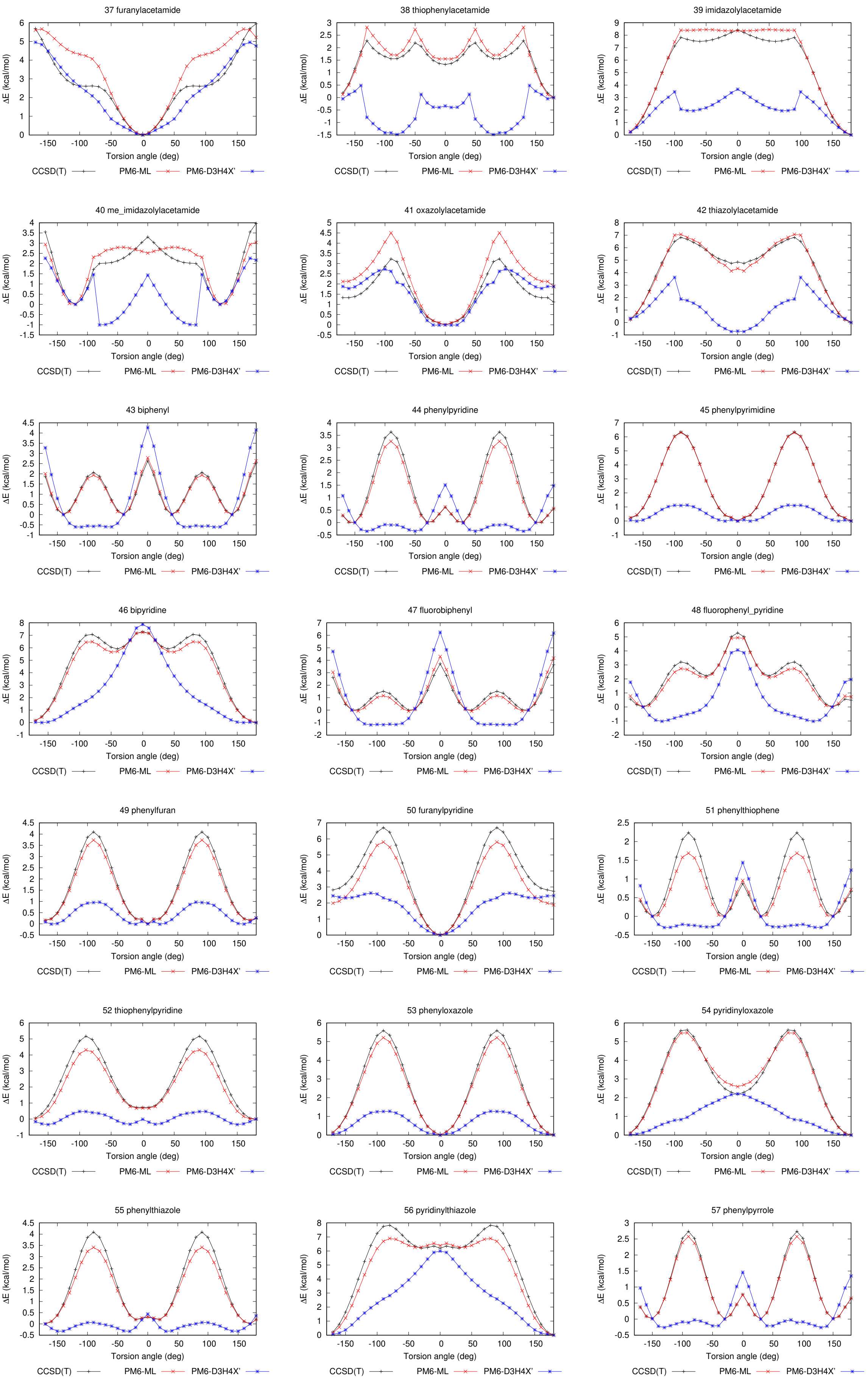
37 furanylacetamide
38 thiophenylacetamide
39 imidazolylacetamide
40 me_imidazolylacetamide
41 oxazolylacetamide
42 thiazolylacetamide
43 biphenyl
44 phenylpyridine
45 phenylpyrimidine
46 bipyridine
47 fluorobiphenyl
48 fluorophenyl_pyridine
49 phenylfuran
50 furanylpyridine
51 phenylthiophene
52 thiophenylpyridine
53 phenyloxazole
54 pyridinyloxazole
55 phenylthiazole
56 pyridinylthiazole
57 phenylpyrrole
ΔE (kcal/mol)
Torsion angle (deg)
CCSD(T)
PM6-ML
PM6-D3H4X'

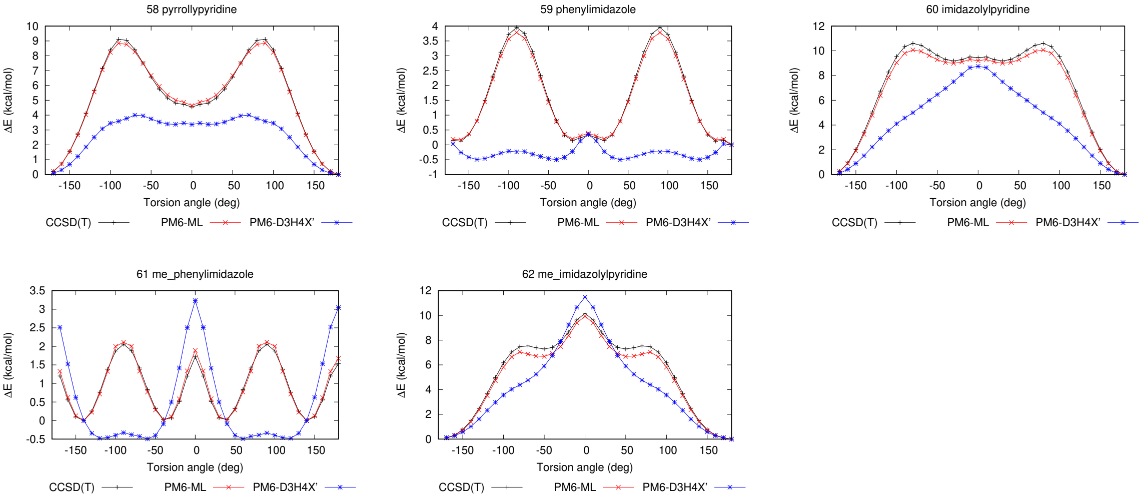
58 pyrrollypyridine
59 phenylimidazole
60 imidazolylpyridine
61 me_phenylimidazole
62 me_imidazolylpyridine
ΔE (kcal/mol)
Torsion angle (deg)
CCSD(T)
PM6-ML
PM6-D3H4X'
